# Hydrazides in the reaction with hydroxypyrrolines: less nucleophilicity – more diversity

**DOI:** 10.3762/bjoc.17.29

**Published:** 2021-01-29

**Authors:** Dmitrii A Shabalin, Evgeniya E Ivanova, Igor A Ushakov, Elena Yu Schmidt, Boris A Trofimov

**Affiliations:** 1 A. E. Favorsky Irkutsk Institute of Chemistry, Siberian Branch of the Russian Academy of Sciences, 1 Favorsky St, 664033 Irkutsk, Russian Federation

**Keywords:** hydrazides, pyridazines, pyrrolines, recyclization, ring expansion

## Abstract

Expedient protocols for the synthesis of three types of highly functionalized azaheterocyclic scaffolds (dihydropyridazines, tetrahydropyridazines, and partially saturated tricyclic systems) from readily available hydroxypyrrolines and hydrazides are described. The directions of the transformation of a common initial intermediate, namely a Brønsted acid-activated hydroxypyrroline, depend on the reaction conditions and the structure of the hydrazides.

## Introduction

**Figure 1:**
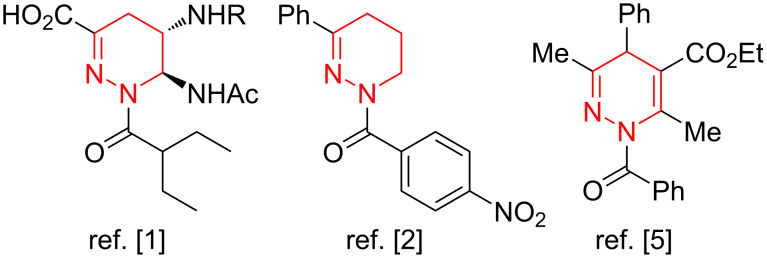
Biologically active di- and tetrahydropyridazines.

Di- and tetrahydropyridazines are valuable heterocyclic motifs which are utilized as key fragments of influenza neuraminidase inhibitors [1], nonsteroidal progesterone receptor regulators [2–3], anti-inflammatory [4], antihypertensive and spasmolytic [5–7] agents (Figure 1). It is due to their prospects in drug design that a search for effective synthetic protocols to construct partially saturated pyridazine scaffolds is both highly topical and necessary.

**Scheme 1:**
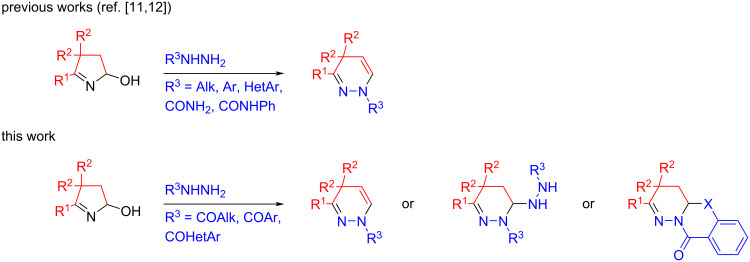
Recyclization of hydroxypyrrolines with hydrazine derivatives.

Recently, we have developed a convenient approach to the 1,4-dihydropyridazine core based on the Brønsted acid-catalyzed regioselective recyclization of 5-hydroxypyrrolines (assembled in a one-pot manner from ketoximes and acetylene gas [8–9] or calcium carbide [10]) with hydrazines (Scheme 1) [11–12]. The reaction is operationally simple and tolerates a wide range of hydrazines including alkyl-, aryl-, and hetarylhydrazines [11] as well as semicarbazide and its derivatives [12].

However, the scope of this method with a special emphasis on the nature of hydrazine derivatives still remains obscure. Meeting this challenge becomes particularly important in view of the significance of the less nucleophilic hydrazides as N–N-bond containing starting materials in drug discovery [13–16].

Herein, we report how a lower nucleophilicity of the hydrazides diversifies the recyclization of 5-hydroxypyrrolines and results in the chemoselective formation of highly functionalized azaheterocyclic scaffolds. Such a short-cut to partially saturated azaheterocyclic scaffolds meets the interests of modern medicinal chemistry which today focuses on the study of partially saturated molecules because sp^3^ carbon centers make the molecules potentially chiral and 3D structured, which improves the odds for drug discovery [17].

## Results and Discussion

On the basis of our previous works [11–12] the reaction of 3,3-dimethyl-5-hydroxy-2-phenyl-Δ^1^-pyrroline (**1a**) with an equimolar amount of benzohydrazide (**2a**) was initially performed in the presence of trifluoroacetic acid (TFA, 10 mol %) in refluxing acetonitrile for 3 h. Unexpectedly, 6-(2'-benzoylhydrazinyl)-1,4,5,6-tetrahydropyridazine **3aa** was isolated in a 46% yield, while the anticipated 1,4-dihydropyridazine **4aa** was not detected at all (Table 1, entry 1). Although the increase of the reaction time up to 5 h slightly improved the yield of tetrahydropyridazine **3aa** (53%, entry 2 in Table 1), more efficient appeared to be the use of a two-fold excess of benzohydrazide (**2a**) that provided **3aa** in 93% isolated yield after reflux for 3 h (Table 1, entry 4). With these optimal conditions for the synthesis of tetrahydropyridazine **3aa** in hand, we next turned our attention to its transformation to 1,4-dihydropyridazine **4aa**. Both the prolonged reflux for 6 h (Table 1, entry 5) and the addition of 100 mol % of TFA (Table 1, entry 6) did not significantly promote the **3aa**→**4aa** transformation, that was apparently caused by the deactivation of the acid catalyst by ammonia, released in the preceding step (vide infra for the mechanism discussion). To prevent this process, a stepwise addition of TFA was tested (Table 1, entries 7–11). Finally, the best isolated yield of 1,4-dihydropyridazine **4aa** (74%) was achieved by a two-step, one-pot protocol, comprising the initial recyclization of hydroxypyrroline **1a** with 2 equiv of benzohydrazide (**2a**) in refluxing acetonitrile for 3 h in the presence of 10 mol % of TFA followed by the introduction of 140 mol % of TFA and additional reflux for 3 h (Table 1, entry 11).

**Table 1:** A study of the model reaction between hydroxypyrroline **1a** and benzohydrazide (**2a**)^a^.

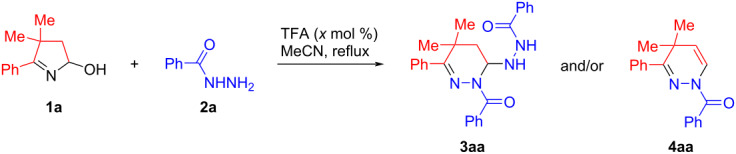				

entry	TFA loading (*x* mol %)	time (h)	yield of **3aa**^b^	yield of **4aa**^b^

1	10	3	46	0
2	10	5	53	0
3	10	1	33	0
4	10	3	93	0
5	10	6	92	0
6	100	6	**3aa**:**4aa** = 78:22^c^	
7	10 + 40	3 + 3	**3aa**:**4aa** = 55:45^c^	
8	10 + 90	3 + 3	**3aa**:**4aa** = 28:72^c^	
9	10 + 90	3 + 4	**3aa**:**4aa** = 22:78^c^	
10	10 + 90	3 + 5	**3aa**:**4aa** = 22:78^c^	
11	10 + 140	3 + 3	**3aa**:**4aa** = 3:97 (74%)^b,c^	

^a^Reaction conditions: hydroxypyrroline **1a** (95 mg, 0.5 mmol), benzohydrazide (**2a**, 0.5 mmol for entries 1 and 2, 1.0 mmol for entries 3–11), TFA (*x* mol %), acetonitrile (3 mL), reflux; ^b^if not stated otherwise, isolated yields are given; ^c^according to ^1^H NMR of the crude reaction mixture.

**Scheme 2:**
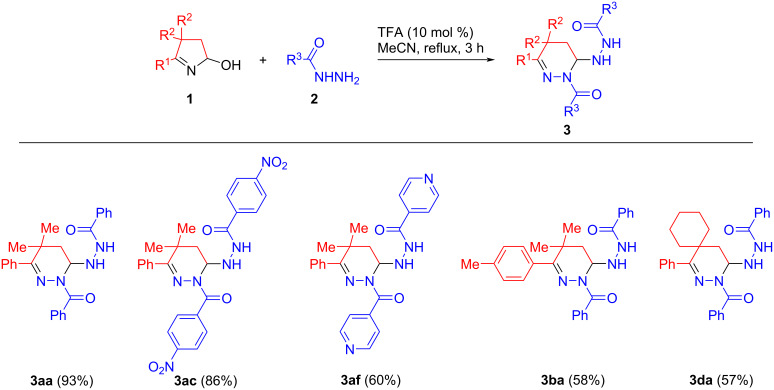
Synthesis of tetrahydropyridazines **3**.

Next, the substrate scope and functional group tolerance of the protocols aimed to the chemoselective synthesis of di- and tetrahydropyridazines were investigated. Several features of the developed methods are noteworthy. It was found that all tested hydroxypyrrolines **1a**–**e** and hydrazides **2a**–**g** afforded the corresponding tetrahydropyridazines **3** (according to ^1^H NMR analysis of the crude reaction mixtures). However, due to the difficulties associated with their isolation and purification only few examples of analytically pure samples were obtained in 57–93% isolated yields (Scheme 2).

**Scheme 3:**
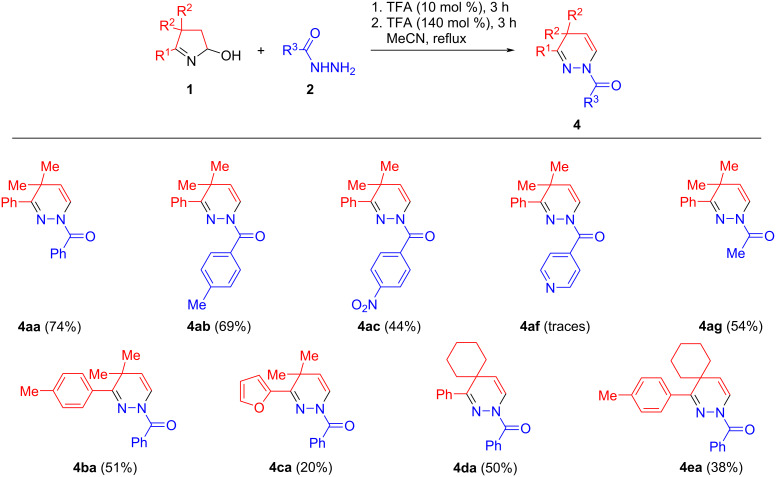
Synthesis of dihydropyridazines **4**.

When the same set of hydroxypyrrolines **1a**–**e** and hydrazides **2a**–**g** was subjected to the two-step, one-pot protocol, the desired 1,4-dihydropyridazines **4** were formed in 20–74% isolated yields (Scheme 3). Electron-rich hydrazides appeared to be good recyclization agents as was exemplified by the assembly of 1,4-dihydropyridazines **4ab** and **4ag** from 4-methylbenzohydrazide (**2b**) and acetohydrazide (**2g**) in 69% and 54% isolated yields, respectively. In contrast, the recyclization of hydroxypyrroline **1a** with electron-deficient 4-nitrobenzohydrazide (**2c**) proceeded somewhat less efficiently to afford the corresponding 1,4-dihydropyridazine **4ac** in 44% isolated yield. The structural fragment of the well-known antibiotic isoniazid (**2f**) was successfully incorporated in the tetrahydropyridazine core (the yield of **3af** was 60%, Scheme 2), but only traces of the 1,4-dihydropyridazine **4af** were detected following the two-step, one-pot protocol (Scheme 3). Apparently, the basic pyridine nitrogen atoms of intermediate **3af** deactivate the acid catalyst, whilst the use of 3.4 equiv of TFA causes the full protonation of the pyridine rings thus making them strongly electron-deficient substituents that finally prevent an acid-catalyzed elimination of hydrazide **2f** (vide infra for the mechanism discussion). Finally, the diverse hydroxypyrrolines **1b**–**e** were tested in the studied reaction and the corresponding 1,4-dihydropyridazines **4ba**–**ea** were obtained in 20–51% isolated yields.

**Scheme 4:**
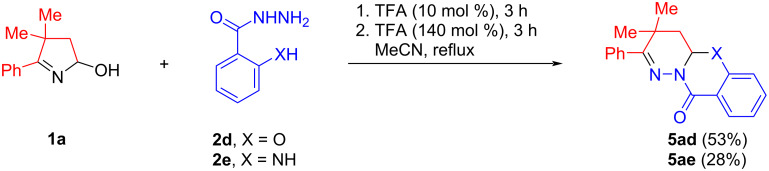
Recyclization of hydroxypyrroline **1a** with hydrazides **2d** and **2e**.

Interestingly, when hydroxypyrroline **1a** reacted under a two-step, one-pot protocol with the hydrazides of salicylic (**2d**) and anthranilic (**2e**) acids having additional nucleophilic functions, the corresponding tricycles **5ad** and **5ae** were formed in 53% and 28% isolated yields, respectively (Scheme 4).

**Scheme 5:**
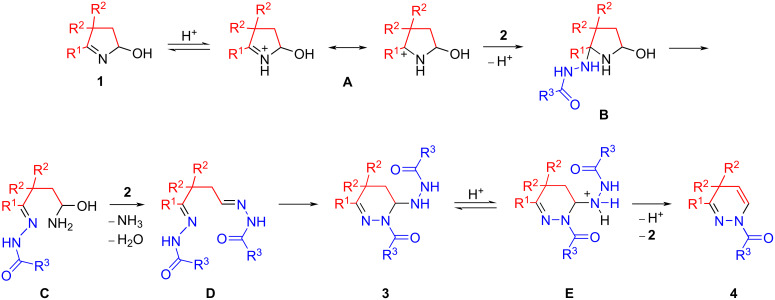
A proposed mechanism for the recyclization of hydroxypyrrolines **1** with hydrazides **2**.

A proposed mechanism for the recyclization of hydroxypyrrolines **1** with hydrazides **2** is shown in Scheme 5. The protonation of the starting hydroxypyrroline **1** with TFA leads to the formation of cation **A**, which reacts with hydrazide **2** to give the pyrrolidine derivative **B**. The latter undergoes a ring opening with formation of the linear intermediate **C**. In comparison with alkyl-, aryl-, and hetarylhydrazines [11] as well as semicarbazide and its derivatives [12], the nucleophilicity of the internal nitrogen atom of hydrazide **2** is significantly reduced. As a result, the interception of the intermediate **C** by the second molecule of hydrazide **2** affords dihydrazone **D** (similar to previously observed in the recyclization with 2,4-dinitrophenylhydrazine [11]), which further cyclizes to tetrahydropyridazine **3**. An acid-catalyzed elimination of hydrazide **2** from tetrahydropyridazine **3** completes the formation of 1,4-dihydropyridazine **4**.

## Conclusion

In summary, an acid-catalyzed recyclization of hydroxypyrrolines (easily synthesized from ketoximes and acetylene) with hydrazides was studied. The reduced nucleophilicity of the hydrazides in comparison with other hydrazine derivatives constitutes the basis for the convenient chemoselective syntheses of highly functionalized azaheterocyclic scaffolds (di- and tetrahydropyridazines) with an acyl function at the nitrogen atom, thus having a substituent pattern similar to that of the known biologically active compounds of the pyridazine family. Moreover, the presence of other nucleophilic functions in the hydrazide component creates diversification points and enables more complex molecular architectures to be assembled that was demonstrated by the synthesis of partially saturated azatricyclic systems.

## Supporting Information

File 1 Experimental methods, compound characterization data, and copies of ^1^H and ^13^C NMR spectra.
