# Supplementary material for: Hydrazides in the reaction with hydroxypyrrolines: less nucleophilicity – more diversity
Source: Beilstein J Org Chem. 2021 Jan 29;17:319–24. doi: 10.3762/bjoc.17.29 (PMC7849252; doi:10.3762/bjoc.17.29)
Supplement: File 1 — Experimental methods, compound characterization data, and copies of 1H and 13C NMR spectra. [file Beilstein_J_Org_Chem-17-319-s001.pdf]

BEILSTEIN JOURNAL OF ORGANIC CHEMISTRY

Supporting Information

for

Hydrazides in the reaction with hydroxypyrrolines: less nucleophilicity – more diversity

Dmitrii A. Shabalin, Evgeniya E. Ivanova, Igor A. Ushakov, Elena Yu. Schmidt and Boris A. Trofimov

Beilstein J. Org. Chem. **2021,** *17,* 319–324. doi:10.3762/bjoc.17.29

Experimental methods, compound characterization data, and copies of ^{1}H and ^{13}C NMR spectra

License and Terms: This is a supporting information file under the terms of the Creative Commons Attribution License (https://creativecommons.org/licenses/by/4.0). Please note that the reuse, redistribution and reproduction in particular requires that the author(s) and source are credited and that individual graphics may be subject to special legal provisions.
The license is subject to the *Beilstein Journal of Organic Chemistry* terms and conditions: (https://www.beilstein-journals.org/bjoc/terms)

Table of contents

General information S2

General procedure for the synthesis of 1,4,5,6-tetrahydropyridazines **3** S3

^{1}H and ^{13}C NMR spectra of 1,4,5,6-tetrahydropyridazines **3**... S5

General procedure for the synthesis of 1,4-dihydropyridazines **4** and tricycles **5** S10

^{1}H and ^{13}C NMR spectra of 1,4-dihydropyridazines **4** and tricycles **5**......................................S13

General information

All chemicals and solvents were purchased from commercial sources and used without further purification. Commercial acetonitrile was dried with 4 Å MS before use. The starting 5-hydroxy-Δ^1-pyrrolines **1a–e** were prepared by a literature method.[1] Thin layer chromatography was carried out on Merck silica gel 60 F_{254} precoated aluminum foil sheets and were visualized using UV light (254 nm). Column chromatography was carried out using slurry packed Sigma Aldrich silica gel (SiO_2), 70–230 mesh, pore size 60 Å, eluent – hexane/diethyl ether 1:1 (v/v). NMR spectra were recorded from solutions in $CDCl_3$ or DMSO-d_6 on Bruker DPX-400 and AV-400 spectrometers (400.1 MHz for ^{1}H and 100.6 MHz for ^{13}C). Chemical shifts (δ) are quoted in parts per million (ppm). The residual solvent peak, δ_H 7.27 and δ_C 77.10 for $CDCl_3$, δ_H 2.50 and δ_C 39.50 for DMSO-d_6, was used as a reference. Coupling constants (*J*) are reported in hertz (Hz). The multiplicity abbreviations used are: s singlet, d doublet, dd doublet of doublet, t triplet, q quartet, m multiplet, br broad signal. Signals were assigned through analysis of 2D COSY, NOESY, HMBC, and HSQC experiments, if required. High-resolution mass spectra were recorded from acetonitrile solutions with 0.1% HFBA on HPLC Agilent 1200/Agilent 6210 TOF instrument equipped with an electrospray ionization (ESI) source. Melting points (uncorrected) were measured on a digital melting point apparatus Electrothermal IA 9200.

---

[1] a) D. A. Shabalin, M. Yu. Dvorko, E. Yu. Schmidt, I. A. Ushakov, B. A. Trofimov, *Tetrahedron* **2016**, *72*, 6661-6667; b) D. A. Shabalin, M. Yu. Dvorko, E. Yu. Schmidt, N. I. Protsuk, B. A. Trofimov, *Tetrahedron Lett.* **2016**, *57*, 3156-3159.

General procedure for the synthesis of 1,4,5,6-tetrahydropyridazines 3

A mixture of 5-hydroxy-Δ^1-hydroxypyrroline **1** (0.5 mmol), hydrazide **2** (1.0 mmol), acetonitrile (3 mL), and trifluoroacetic acid (4 µL, 0.05 mmol, 10 mol %) was placed in a 10-mL round-bottomed flask with a stirring bar, equipped with reflux condenser, and heated for 3 h at 80 °C (silicon oil bath). The residue after solvent evaporation was treated with diethyl ether or diethyl ether/hexane 1:1 (v/v) mixture to afford after filtration the desired 1,4,5,6-tetrahydropyridazine **3** as a powder.

N'-(2-Benzoyl-5,5-dimethyl-6-phenyl-2,3,4,5-tetrahydropyridazin-3-yl)benzohydrazide (**3aa**). Following the general procedure, **3aa** was prepared from 3,3-dimethyl-5-hydroxy-2-phenyl-Δ^1-pyrroline (**1a**, 95 mg, 0.5 mmol) and benzohydrazide (**2a**, 136 mg, 1.0 mmol); **3aa** was isolated as a white powder (199 mg, 93% yield), mp 162-164 °C. ^{1}H NMR (400.1 MHz, DMSO-d_6): δ = 10.02 (d, *J* = 5.8 Hz, 1H, NH), 7.80 (d, *J* = 7.5 Hz, 2H, Ph), 7.55-7.52 (m, 3H, Ph), 7.47-7.45 (m, 2H, Ph), 7.35-7.31 (m, 8H, Ph), 5.97-5.95 (m, 1H, NH), 5.86 (dd, *J* = 6.2 Hz, *J* = 4.5 Hz, 1H, CH), 2.29 (dd, *J* = 14.4 Hz, *J* = 4.5 Hz, 1H, CH_2), 2.08 (dd, *J* = 14.4 Hz, *J* = 6.2 Hz, 1H, CH_2), 1.43 (s, 3H, Me), 1.16 (s, 3H, Me). ^{13}C NMR (100.6 MHz, $CDCl_3$): δ = 172.0, 166.5, 158.4, 137.1, 134.9, 132.7, 131.8, 130.5, 130.0, 128.7, 128.4, 128.1, 128.0, 127.4, 127.1, 66.9, 39.1, 32.0, 28.3, 27.6. HRMS (ESI-TOF) calcd for $[C_{26}H_{26}N_4O_2+H]^+$ 427.2134, found 427.2137.

N'-(5,5-Dimethyl-2-(4-nitrobenzoyl)-6-phenyl-2,3,4,5-tetrahydropyridazin-3-yl)-4-nitrobenzohydrazide (**3ac**). Following the general procedure, **3ac** was prepared from 3,3-dimethyl-5-hydroxy-2-phenyl-Δ^1-pyrroline (**1a**, 95 mg, 0.5 mmol) and 4-nitrobenzohydrazide (**2c**, 181 mg, 1.0 mmol); **3ac** was isolated as a white powder (222 mg, 86% yield), mp 201-203 °C. ^{1}H NMR (400.1 MHz, DMSO-d_6): δ = 10.44 (d, *J* = 5.7 Hz, 1H, NH), 8.32 (d, *J* = 8.8 Hz, 2H, Ar), 8.20 (d, *J* = 8.6 Hz, 2H, Ar), 8.06 (d, *J* = 8.8 Hz, 2H, Ar), 7.80 (d, *J* = 8.6 Hz, 2H, Ar), 7.33-7.29 (m, 5H, Ph), 6.06 (m, 1H, NH), 5.95 (dd, *J* = 5.2 Hz, *J* = 3.8 Hz, 1H, CH), 2.32 (dd, *J* = 14.3 Hz, *J* = 3.8 Hz, 1H, CH_2), 2.12 (dd, *J* = 14.3 Hz, *J* = 5.2 Hz, 1H, CH_2), 1.47 (s, 3H, Me), 1.13 (s, 3H, Me). ^{13}C NMR (100.6 MHz, DMSO-d_6): δ = 168.6, 165.0, 159.6, 149.0, 147.6, 142.5, 139.1, 137.1, 129.9, 128.8, 128.2, 128.0, 127.9, 123.5, 122.5, 62.8, 36.7, 31.5, 28.2, 27.6. HRMS (ESI-TOF) calcd for $[C_{26}H_{24}N_6O_6+H]^+$ 517.1836, found 517.1835.

N'-(2-Isonicotinoyl-5,5-dimethyl-6-phenyl-2,3,4,5-tetrahydropyridazin-3-yl)isonicotinohydrazide (**3af**). Following the general procedure, **3af** was prepared from 3,3-dimethyl-5-hydroxy-2-phenyl-Δ^1-pyrroline (**1a**, 95 mg, 0.5 mmol) and isonicotinohydrazide (**2f**,

137 mg, 1.0 mmol); **3af** was isolated as a white powder (128 mg, 60% yield), mp 154-156 °C. ^{1}H NMR (400.1 MHz, DMSO-d$_6$): δ = 10.38 (d, *J* = 4.6 Hz, 1H, NH), 8.73 (d, *J* = 5.6 Hz, 2H, Py), 8.58 (d, *J* = 5.1 Hz, 2H, Py), 7.73 (d, *J* = 5.6 Hz, 2H, Py), 7.46 (d, *J* = 5.1 Hz, 2H, Py), 7.33-7.31 (m, 5H, Ph), 6.02 (m, 1H, NH), 5.91 (dd, *J* = 5.2 Hz, *J* = 3.6 Hz, 1H, CH), 2.29 (dd, *J* = 14.3 Hz, *J* = 3.6 Hz, 1H, CH$_2$), 2.08 (dd, *J* = 14.3 Hz, *J* = 5.2 Hz, 1H, CH$_2$), 1.47 (s, 3H, Me), 1.11 (s, 3H, Me). ^{13}C NMR (100.6 MHz, DMSO-d$_6$): δ = 168.5, 165.0, 159.4, 150.1, 149.0, 143.8, 140.5, 137.1, 128.3, 127.9, 127.9, 122.5, 121.4, 62.6, 36.7, 31.4, 28.3, 27.7. HRMS (ESI-TOF) calcd for $[C_{24}H_{24}N_6O_2+H]^+$ 429.2039, found 429.2041.

N'-(2-Benzoyl-5,5-dimethyl-6-(p-tolyl)-2,3,4,5-tetrahydropyridazin-3-yl)benzohydrazide (**3ba**). Following the general procedure, **3ba** was prepared from 3,3-dimethyl-5-hydroxy-2-(*p*-tolyl)-Δ^1-pyrroline (**1b**, 102 mg, 0.5 mmol) and benzohydrazide (**2a**, 136 mg, 1 mmol); **3ba** was isolated as a cream powder (127 mg, 58% yield), mp 134-136 °C. ^{1}H NMR (400.1 MHz, CDCl$_3$): δ = 8.55 (d, *J* = 5.9 Hz, 1H, NH), 7.82 (d, *J* = 7.6 Hz, 2H, Ph), 7.72 (d, *J* = 8.0 Hz, 2H, Ph), 7.52-7.48 (m, 1H, Ph), 7.44-7.41 (m, 2H, Ph), 7.39-7.36 (m, 1H, Ph), 7.33-7.30 (m, 2H, Ph), 7.26 (d, *J* = 7.8 Hz, 2H, Ar), 7.10 (d, *J* = 7.8 Hz, 2H, Ar), 5.60 (t, *J* = 5.8 Hz, 1H, CH), 2.33 (s, 3H, Me), 2.24-2.22 (m, 2H, CH$_2$), 1.48 (s, 3H, Me), 1.28 (s, 3H, Me). ^{13}C NMR (100.6 MHz, CDCl$_3$): δ = 172.0, 166.5, 158.5, 138.3, 135.0, 134.3, 132.8, 131.7, 130.5, 130.0, 128.7, 128.7, 128.1, 127.4, 127.1, 66.9, 39.2, 32.0, 28.3, 27.6, 21.2. HRMS (ESI-TOF) calcd for$[C_{27}H_{28}N_4O_2+H]^+$ 441.2291, found 441.2291.

N'-(3-Benzoyl-1-phenyl-2,3-diazaspiro[5.5]undec-1-en-4-yl)benzohydrazide (**3da**). Following the general procedure, **3da** was prepared from 1-phenyl-2-azaspiro[4.5]dec-1-en-3-ol (**1d**, 115 mg, 0.5 mmol) and benzohydrazide (**2a** ,136 mg, 1.0 mmol); **3da** was isolated as a cream powder (132 mg, 57% yield), mp 177-178 °C. ^{1}H NMR (400.1 MHz, CDCl$_3$): δ = 8.42 (br s, 1H, NH), 7.81 (d, *J* = 7.5 Hz, 2H, Ph), 7.71 (d, *J* = 7.5 Hz, 2H, Ph), 7.51-7.49 (m, 1H, Ph), 7.45-7.41 (m, 2H, Ph), 7.32-7.27 (m, 8H, Ph), 5.69 (br s, 1H, NH), 5.60 (t, *J* = 6.3 Hz, 1H, CH), 2.47-2.45 (m, 2H, CH$_2$), 1.86-1.82 (m, 1H, Cy), 1.73-1.59 (m, 8H, Cy), 1.21-1.16 (m, 1H, Cy). ^{13}C NMR (100.6 MHz, CDCl$_3$): δ = 171.4, 166.5, 161.2, 136.9, 134.7, 132.7, 131.6, 130.4, 129.9, 128.5, 128.5, 128.1, 127.7, 127.2, 127.1, 66.6, 36.0, 33.6, 33.4, 31.1, 25.4, 21.0, 20.5. HRMS (ESI-TOF) calcd for$[C_{29}H_{30}N_4O_2+H]^+$ 467.2447, found 467.2452.

^{1}H and ^{13}C NMR spectra of 1,4,5,6-tetrahydropyridazines 3

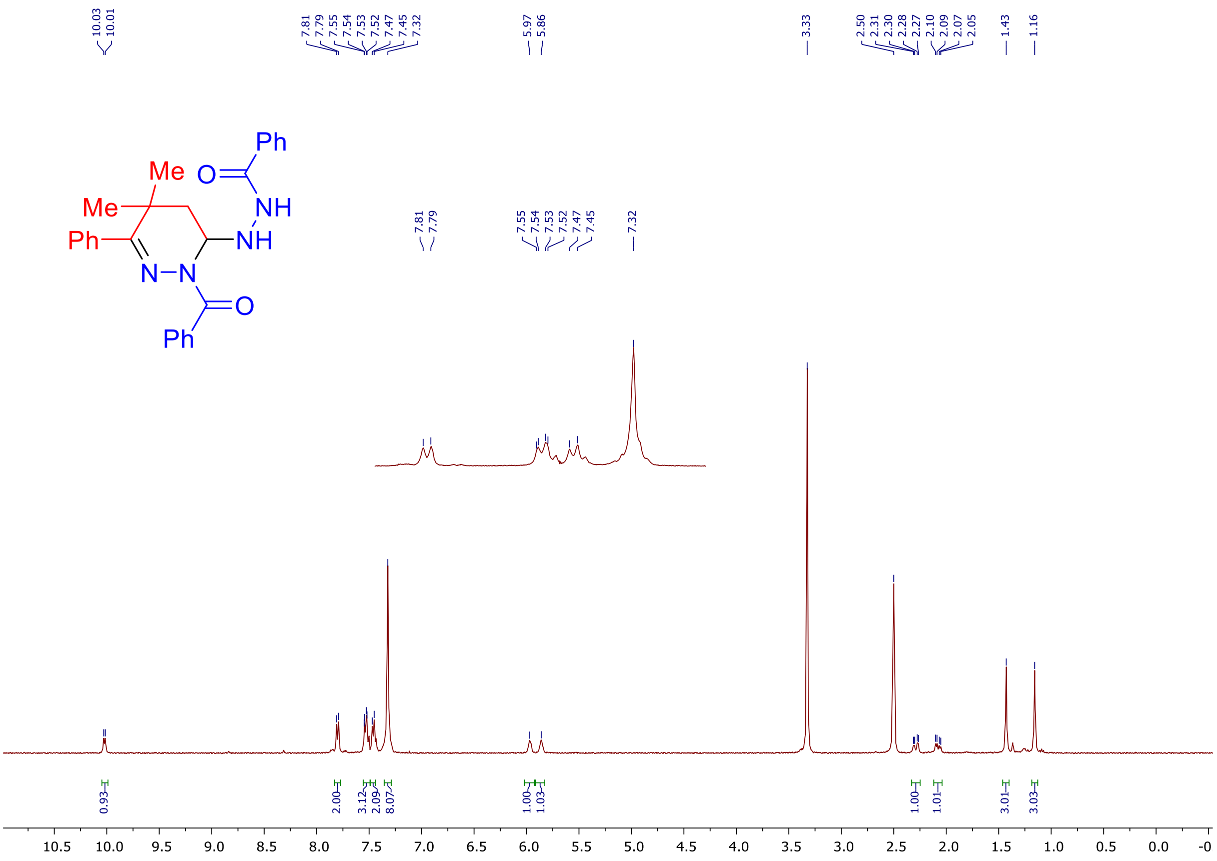

^{1}H NMR spectrum of **3aa** (400.1 MHz, DMSO-d_6)

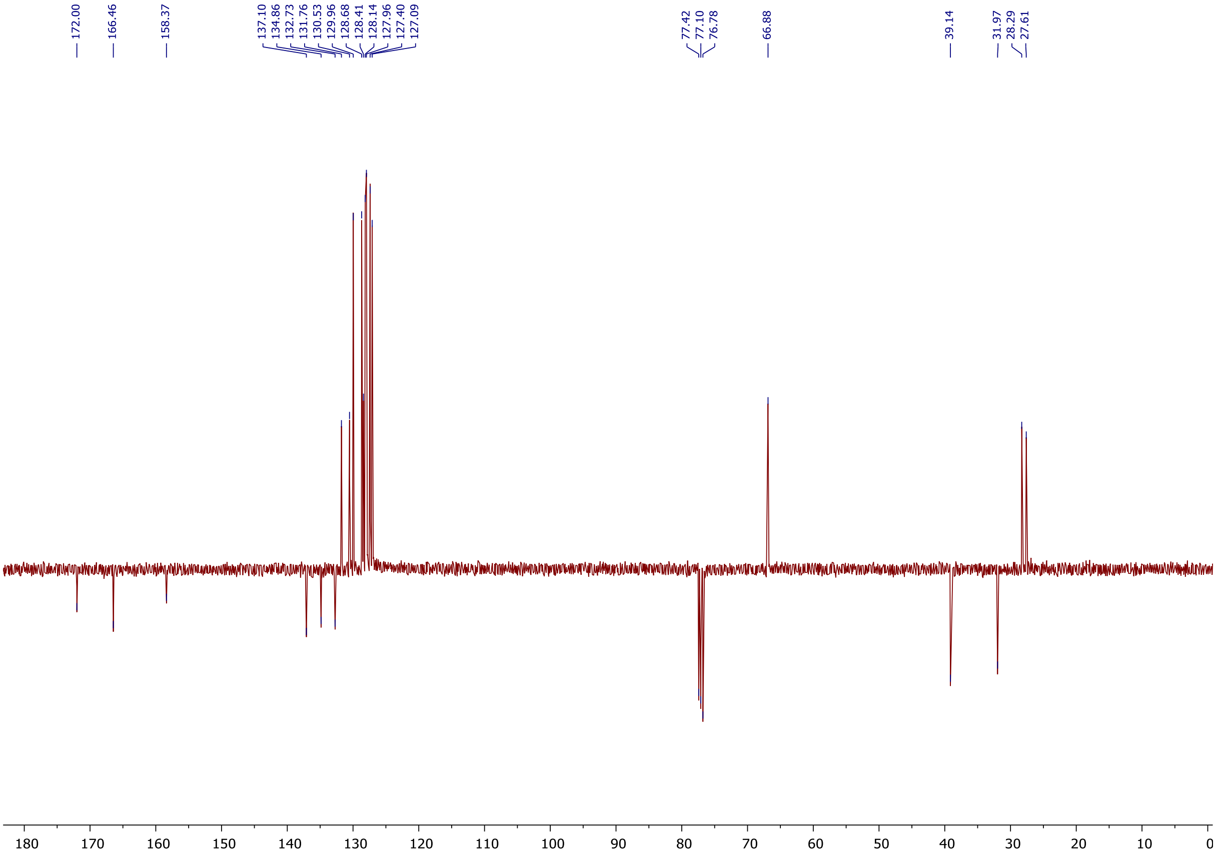

^{13}C jmod NMR spectrum of **3aa** (100.6 MHz, $CDCl_3$)

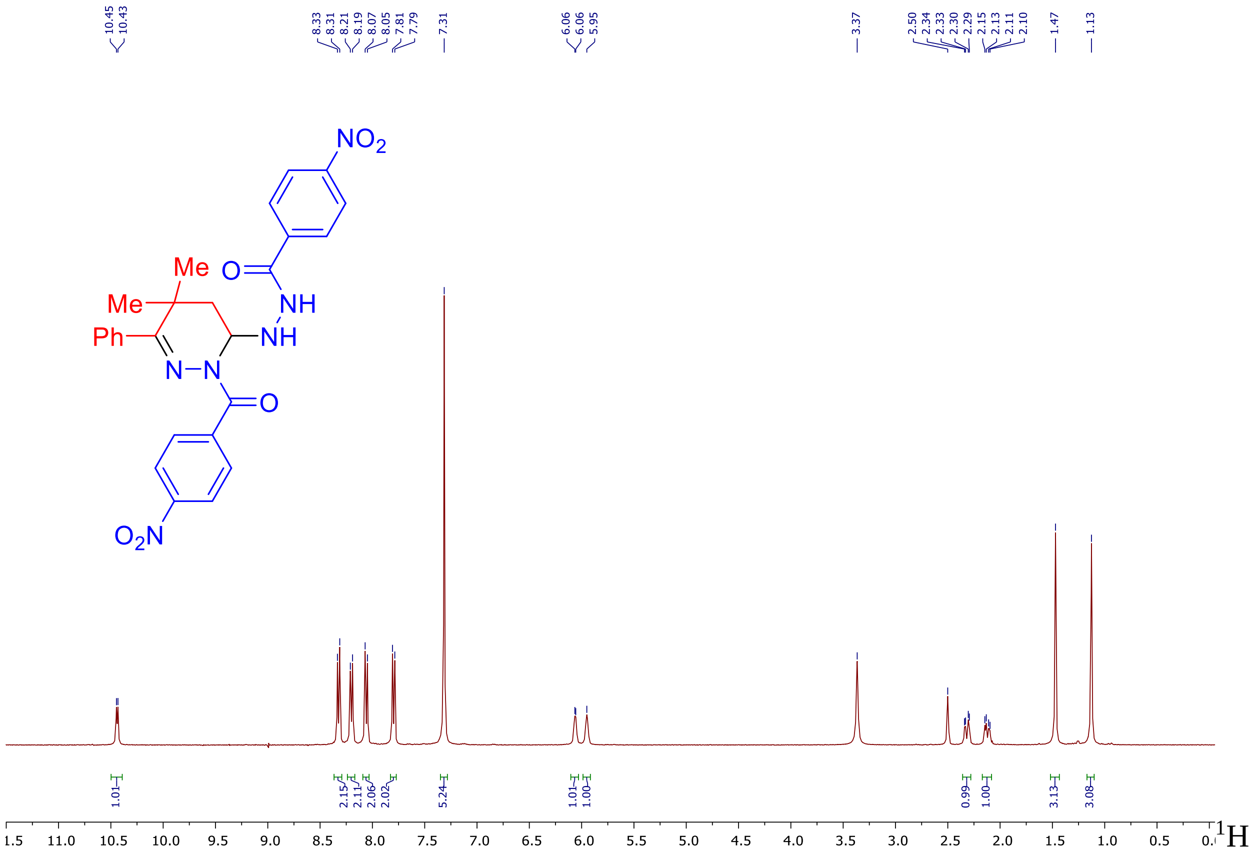

NMR spectrum of **3ac** (400.1 MHz, DMSO-d_6)

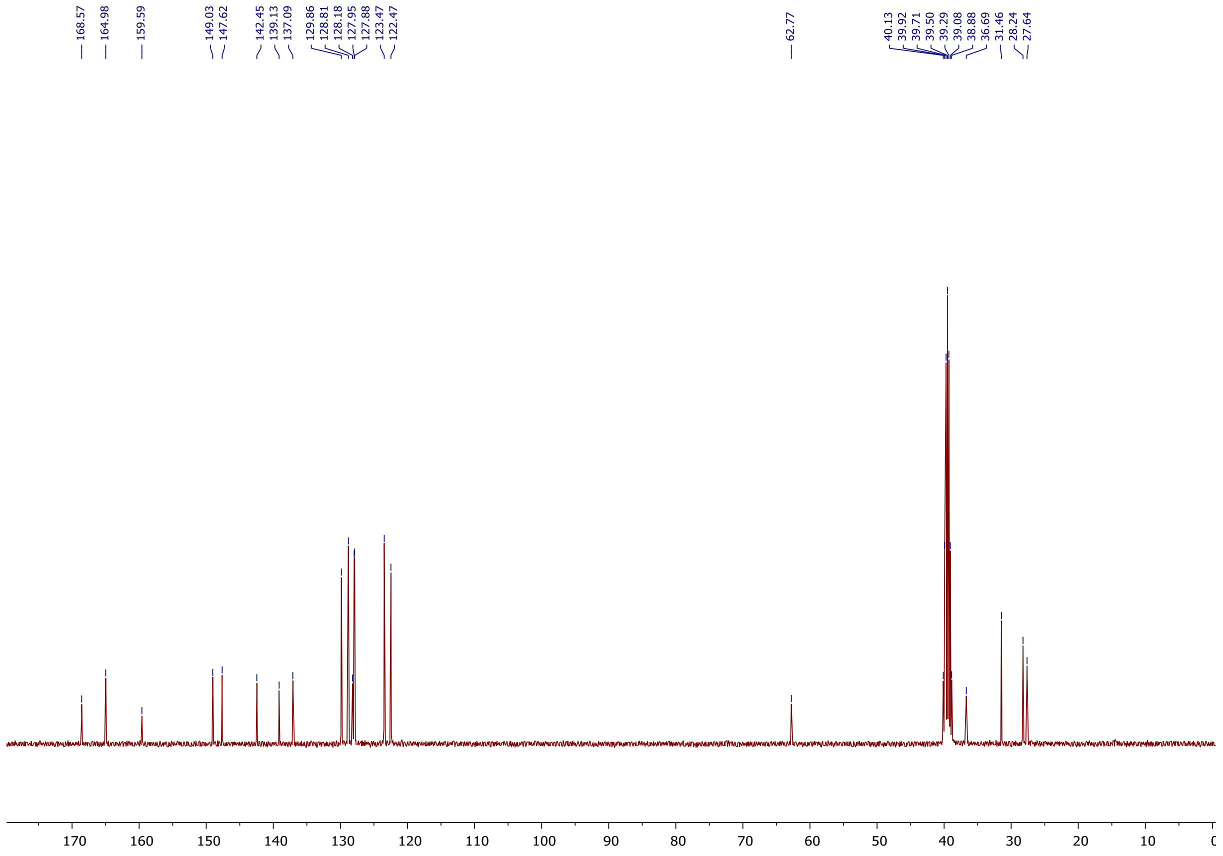

^{13}C NMR spectrum of **3ac** (100.6 MHz, DMSO-d_6)

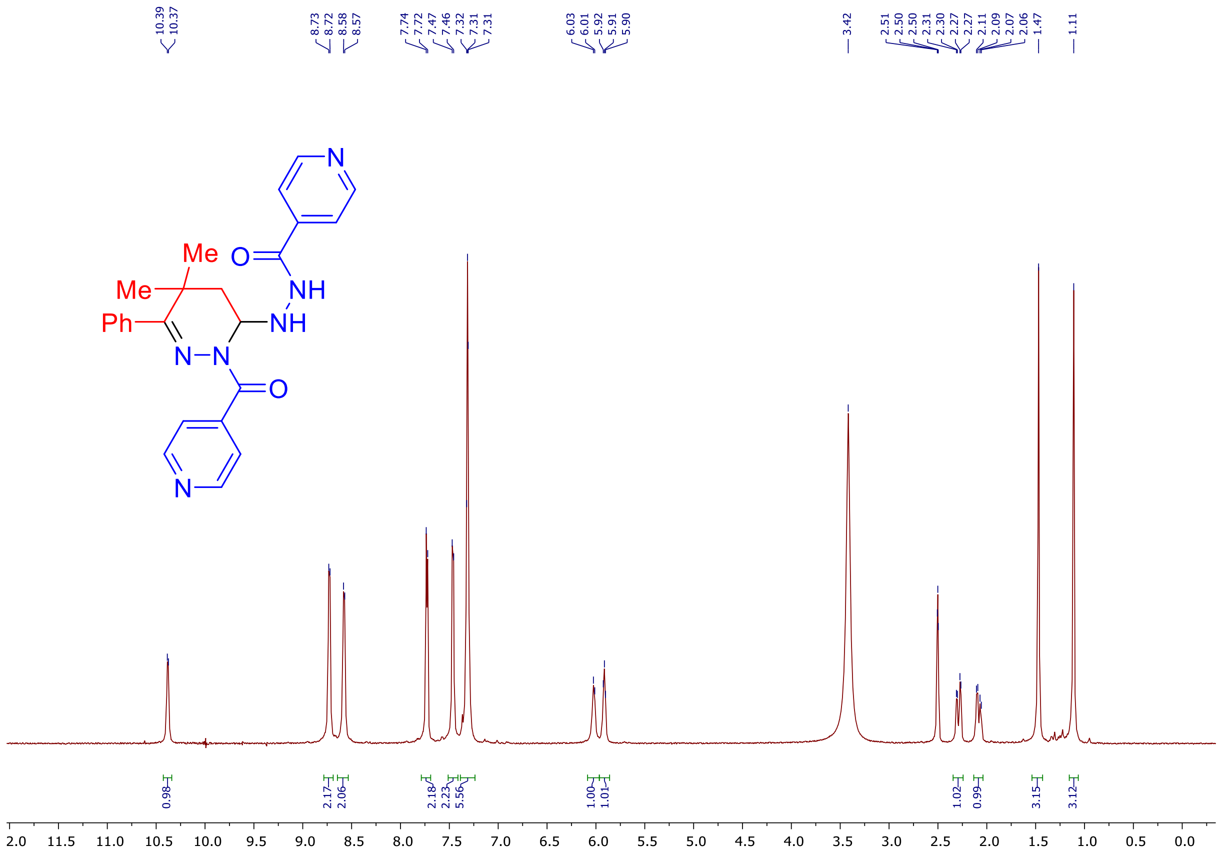

^{1}H NMR spectrum of **3af** (400.1 MHz, DMSO-d_6)

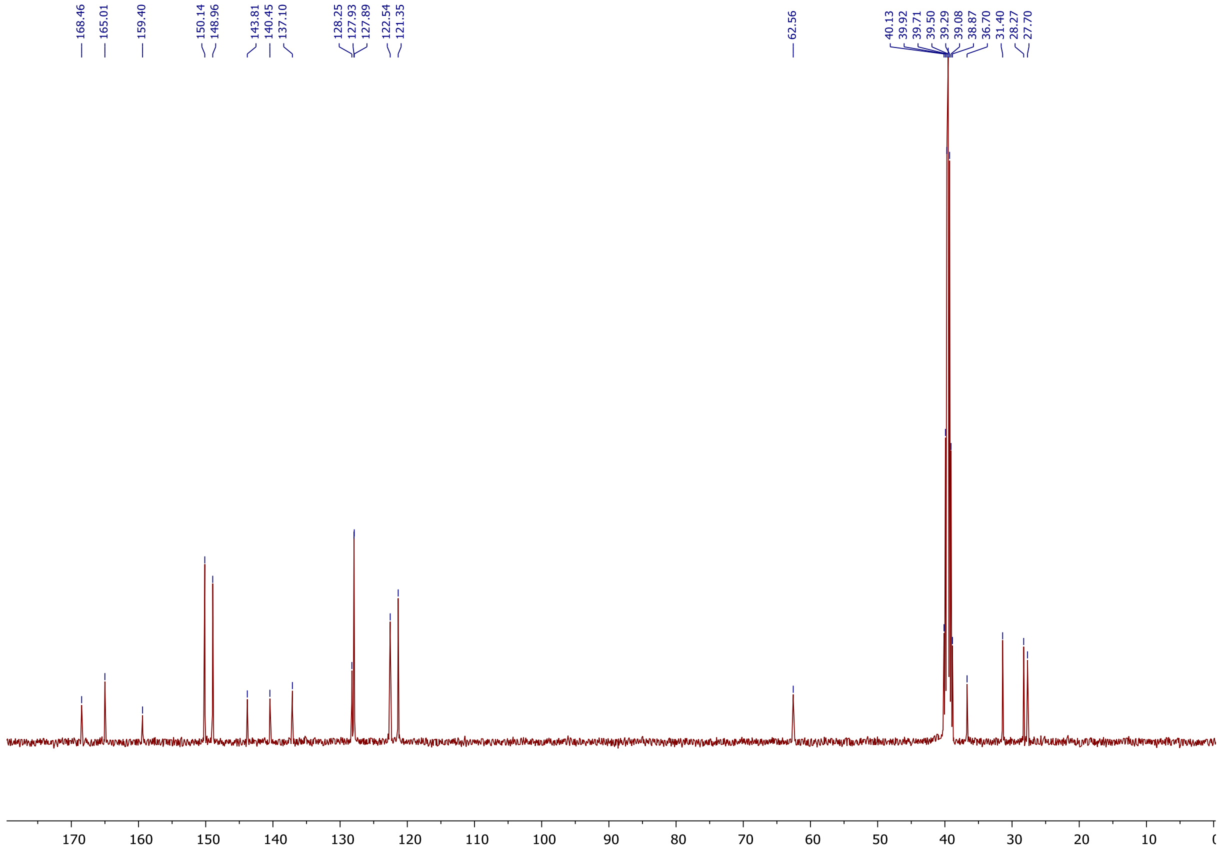

^{13}C NMR spectrum of **3af** (100.6 MHz, DMSO-d_6)

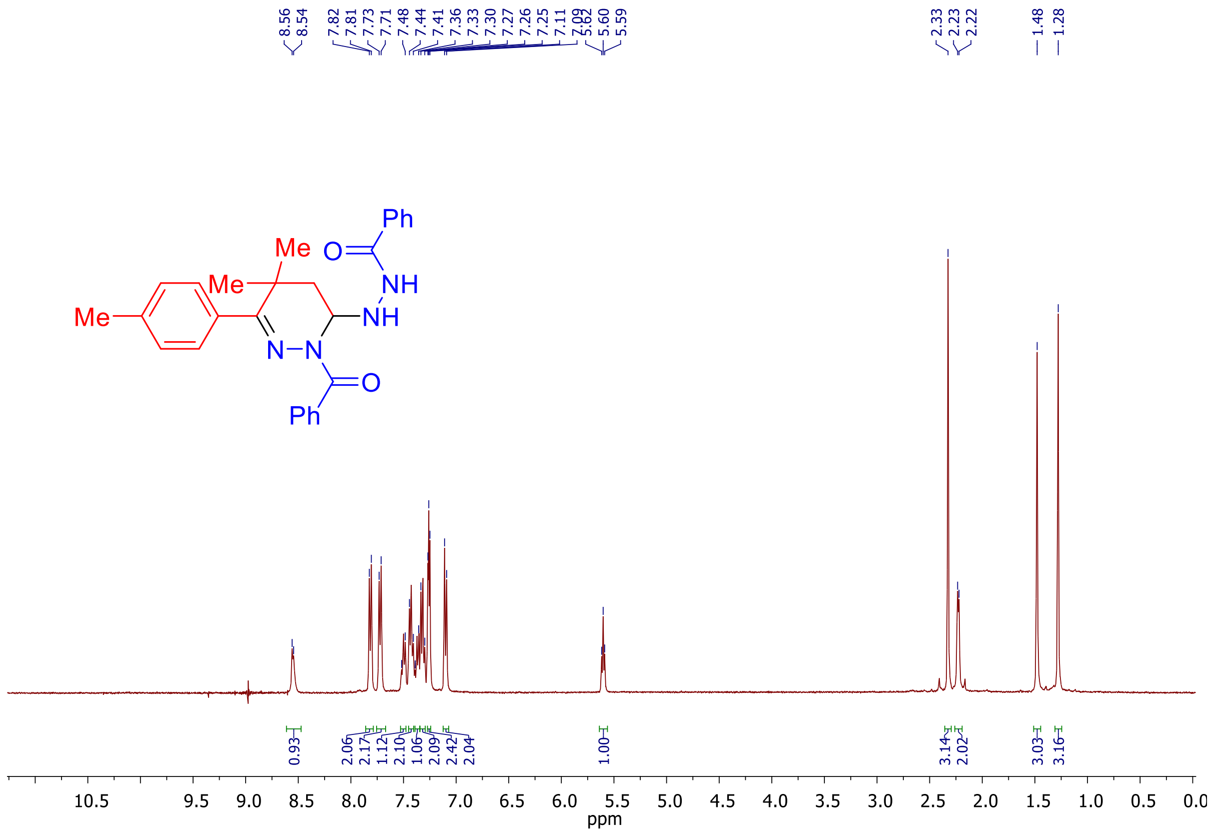

^{1}H NMR spectrum of **3ba** (400.1 MHz, $CDCl_3$)

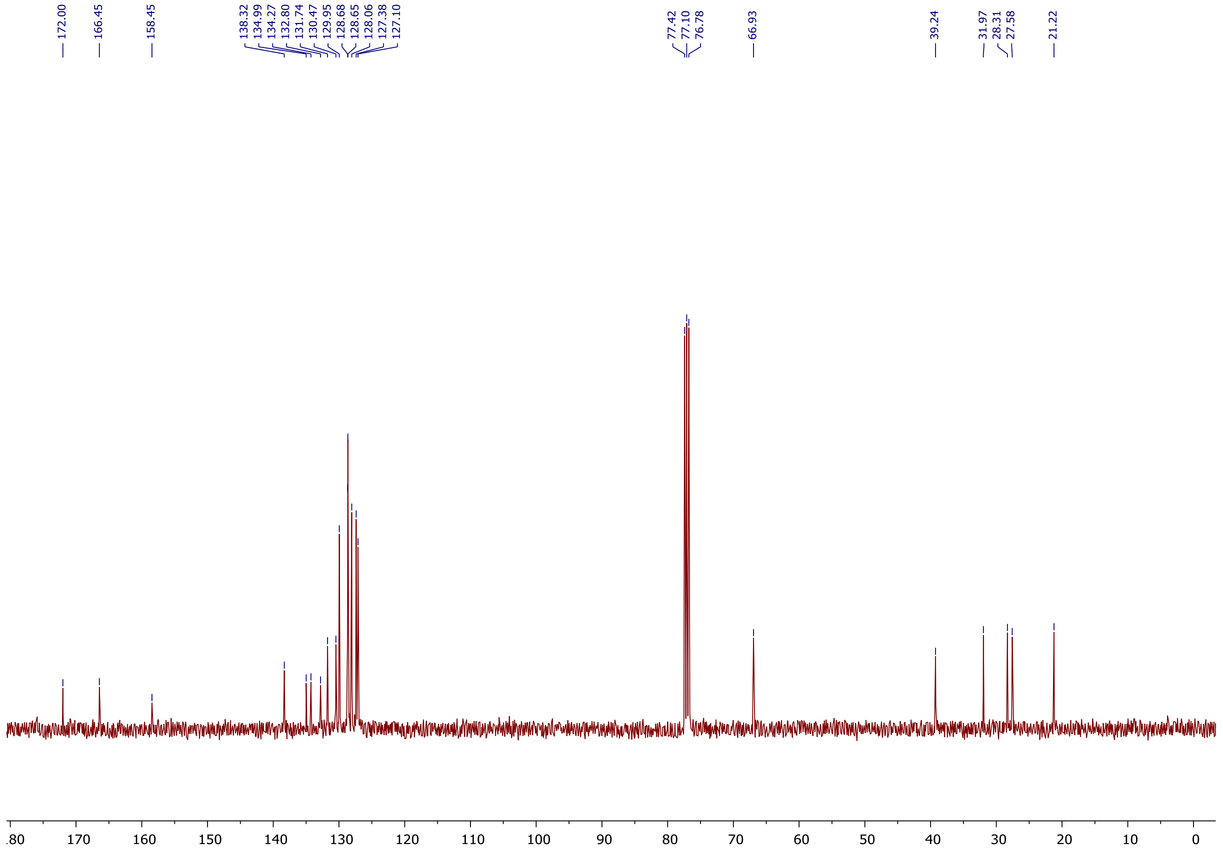

^{13}C NMR spectrum of **3ba** (100.6 MHz, $CDCl_3$)

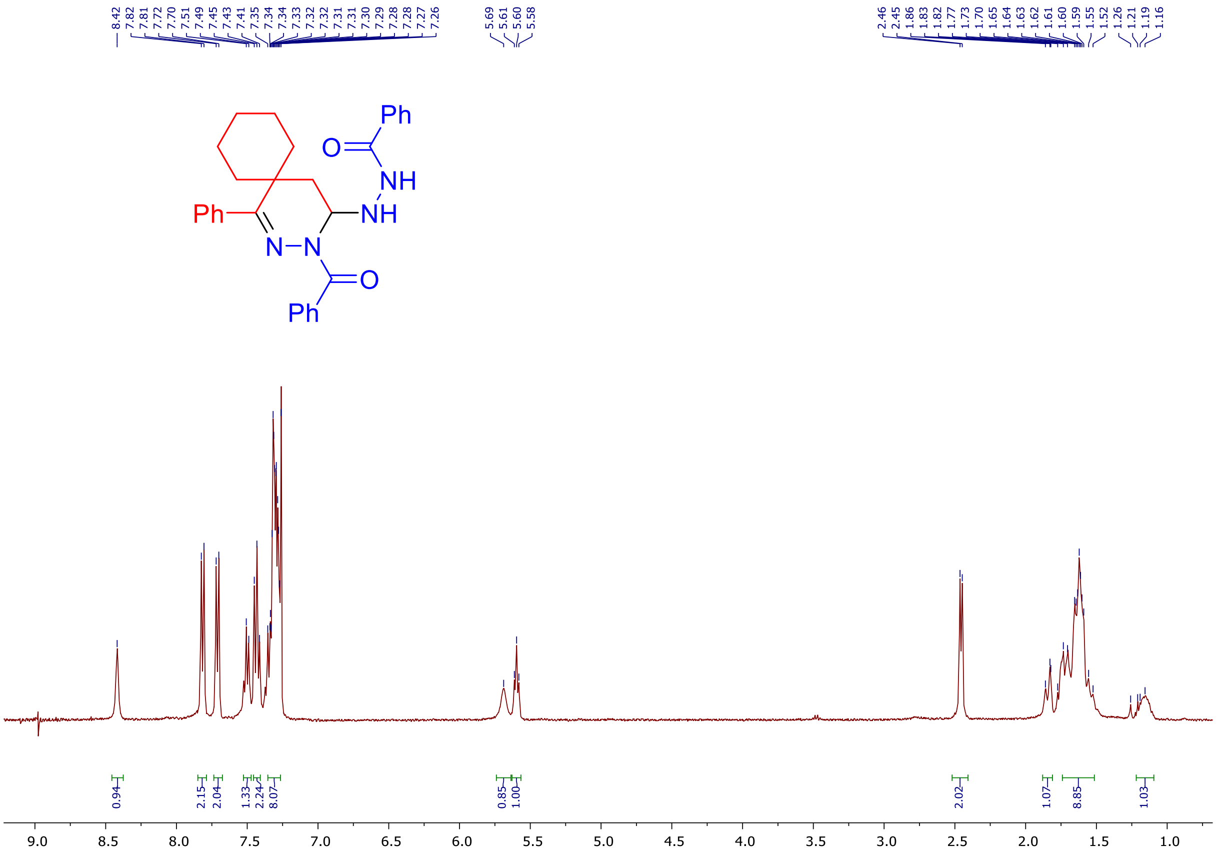

^{1}H NMR spectrum of **3da** (400.1 MHz, $CDCl_3$)

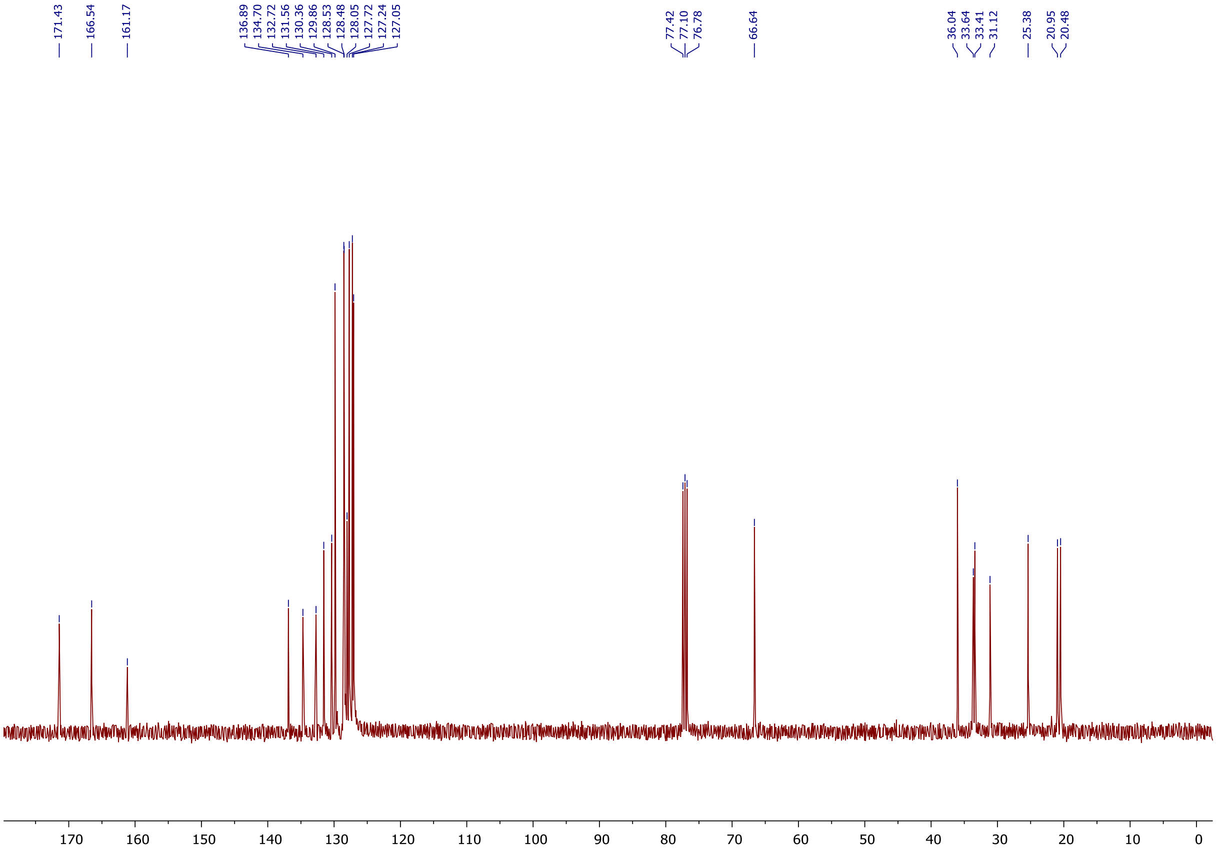

^{13}C NMR spectrum of **3da** (100.6 MHz, $CDCl_3$)

General procedure for the synthesis of 1,4-dihydropyridazines 4 and tricycle 5

A mixture of 5-hydroxy-Δ^1-hydroxypyrroline **1** (0.5 mmol), hydrazide **2** (1.0 mmol), acetonitrile (3 mL), and trifluoroacetic acid (4 μL, 0.05 mmol, 10 mol %) was placed in a 10-mL round-bottomed flask with a stirring bar, equipped with reflux condenser, and heated for 3 h at 80 °C (silicon oil bath). Then, trifluoroacetic acid (54 μL, 0.7 mmol, 140 mol %) was added and the reaction mixture additionally heated for 3 h at 80 °C (silicon oil bath). The residue after solvent evaporation was neutralized with Et_3N (210 μL, 1.5 mmol) and purified by column chromatography on silica to afford the desired 1,4-dihydropyridazine **4** or tricycle **5**.

(4,4-Dimethyl-3-phenylpyridazin-1(4H)-yl)(phenyl)methanone (**4aa**). Following the general procedure, **4aa** was prepared from 3,3-dimethyl-5-hydroxy-2-phenyl-Δ^1-pyrroline (**1a**, 95 mg, 0.5 mmol) and benzohydrazide (**2a**, 136 mg, 1.0 mmol); **4aa** was isolated as a white powder (107 mg, 74% yield), mp 96-98 °C. ^{1}H NMR (400.1 MHz, $CDCl_3$): δ = 7.83-7.80 (m, 2H, Ph), 7.55 (d, *J* = 7.9 Hz, 1H, CH), 7.48-7.46 (m, 2H, Ph), 7.40-7.32 (m, 6H, Ph), 5.13 (d, *J* = 7.9 Hz, 1H, CH), 1.35 (s, 6H, Me). ^{13}C NMR (100.6 MHz, $CDCl_3$): δ = 167.9, 156.1, 136.7, 133.9, 130.8, 130.2, 128.7, 128.7, 127.9, 127.4, 120.1, 116.2, 33.2, 29.3. HRMS (ESI-TOF) calcd for $[C_{19}H_{18}N_2O+H]^+$ 291.1497, found 291.1499.

(4,4-Dimethyl-3-phenylpyridazin-1(4H)-yl)(p-tolyl)methanone (**4ab**). Following the general procedure, **4ab** was prepared from 3,3-dimethyl-5-hydroxy-2-phenyl-Δ^1-pyrroline (**1a**, 95 mg, 0.5 mmol) and 4-methylbenzohydrazide (**2b**, 150 mg, 1.0 mmol); **4ab** was isolated as a cream powder (105 mg, 69% yield), mp 111-113 °C. ^{1}H NMR (400.1 MHz, $CDCl_3$): δ = 7.73 (d, *J* = 7.8 Hz, 2H, Ar), 7.52 (d, *J* = 8.0 Hz, 1H, CH), 7.47-7.45 (m, 2H, Ph), 7.37-7.32 (m, 3H, Ph), 7.14 (d, *J* = 7.8 Hz, 2H, Ar), 5.11 (d, *J* = 8.0 Hz, 1H, CH), 2.34 (s, 3H, Me), 1.33 (s, 6H, Me). ^{13}C NMR (100.6 MHz, $CDCl_3$): δ = 167.8, 156.0, 141.3, 136.9, 130.9, 130.6, 128.8, 128.7, 128.2, 128.0, 120.3, 116.0, 33.2, 29.4, 21.6. HRMS (ESI-TOF) calcd for $[C_{20}H_{20}N_2O+H]^+$ 305.1654, found 305.1654.

(4,4-Dimethyl-3-phenylpyridazin-1(4H)-yl)(4-nitrophenyl)methanone (**4ac**). Following the general procedure, **4ac** was prepared from 3,3-dimethyl-5-hydroxy-2-phenyl-Δ^1-pyrroline (**1a**, 95 mg, 0.5 mmol) and 4-nitrobenzohydrazide (**2c**, 181 mg, 1.0 mmol); **4ac** was isolated as a yellow powder (73 mg, 44% yield), mp 100-102 °C. ^{1}H NMR (400.1 MHz, $CDCl_3$): δ = 8.17 (d, *J* = 8.8 Hz, 2H, Ar), 7.89 (d, *J* = 8.8 Hz, 2H, Ar), 7.50 (d, *J* = 8.1 Hz, 1H, CH), 7.40-7.33 (m, 5H, Ph), 5.20 (d, *J* = 8.1 Hz, 1H, CH), 1.34 (s, 6H, Me). ^{13}C NMR (100.6 MHz, $CDCl_3$): δ =

166.0, 157.6, 148.8, 140.1, 136.3, 131.0, 129.1, 128.5, 128.2, 122.7, 119.3, 117.4, 33.5, 29.4. HRMS (ESI-TOF) calcd for $[C_{19}H_{17}N_3O_3+H]^+$ 336.1348, found 336.1349.

1-(4,4-Dimethyl-3-phenylpyridazin-1(4H)-yl)ethan-1-one (**4ag**). Following the general procedure, **4ag** was prepared from 3,3-dimethyl-5-hydroxy-2-phenyl-Δ^1-pyrroline (**1a**, 95 mg, 0.5 mmol) and acetohydrazide (**2g**, 74 mg, 1.0 mmol); **4ag** was isolated as a light-yellow powder (62 mg, 54% yield), mp 42-44 °C. ^{1}H NMR (400.1 MHz, $CDCl_3$): δ = 7.51-7.48 (m, 2H, Ph), 7.41-7.38 (m, 3H, Ph), 7.34 (d, *J* = 8.1 Hz, 1H, CH), 4.97 (d, *J* = 8.1, Hz, 1H, CH), 2.36 (s, 3H, Me), 1.28 (s, 6H, Me). ^{13}C NMR (100.6 MHz, $CDCl_3$): δ = 170.4, 156.0, 137.1, 128.7, 128.7, 128.0, 118.8, 114.9, 33.0, 29.5, 21.2. HRMS (ESI-TOF) calcd for $[C_{14}H_{16}N_2O+H]^+$ 229.1341, found 229.1340.

(4,4-Dimethyl-3-(p-tolyl)pyridazin-1(4H)-yl)(phenyl)methanone (**4ba**). Following the general procedure, **4ba** was prepared from 3,3-dimethyl-5-hydroxy-2-(*p*-tolyl)-Δ^1-pyrroline (**1b**, 102 mg, 0.5 mmol) and benzohydrazide (**2a**, 136 mg, 1.0 mmol); **4ba** was isolated as a white powder (78 mg, 51% yield), mp 129-131 °C. ^{1}H NMR (400.1 MHz, $CDCl_3$): δ = 7.81 (d, *J* = 8.1 Hz, 2H, Ph), 7.53 (d, *J* = 8.0 Hz, 1H, CH), 7.42-7.33 (m, 3H, Ph, 2H, Ar), 7.14 (d, *J* = 7.8 Hz, 2H, Ar), 5.12 (d, *J* = 8.0 Hz, 1H, CH), 2.35 (s, 3H, Me), 1.35 (s, 6H, Me). ^{13}C NMR (100.6 MHz, $CDCl_3$) δ = 167.9, 156.0, 138.7, 134.0, 133.9, 130.8, 130.3, 128.7, 128.6, 127.4, 120.0, 116.4, 33.2, 29.4, 21.2. HRMS (ESI-TOF) calcd for $[C_{20}H_{20}N_2O+H]^+$ 305.1654, found 305.1654.

(3-(Furan-2-yl)-4,4-dimethylpyridazin-1(4H)-yl)(phenyl)methanone (**4ca**). Following the general procedure, **4ca** was prepared from 3,3-dimethyl-2-(2-furyl)-5-hydroxy-Δ^1-pyrroline (**1c**, 90 mg, 0.5 mmol) and benzohydrazide (**2a**, 136 mg, 1.0 mmol); **4ca** was isolated as an yellow oil (28 mg, 20% yield). ^{1}H NMR (400.1 MHz, $CDCl_3$): δ = 7.82-7.80 (m, 2H, Ph), 7.47-7.39 (m, 1H, CH, 1H, Fur, 3H, Ph), 6.60 (d, *J* = 3.4 Hz, 1H, Fur), 6.39 (dd, *J* = 3.4 Hz, *J* = 1.7 Hz, 1H, Fur), 5.08 (d, *J* = 8.4 Hz, 1H, CH), 1.49 (s, 6H, Me). ^{13}C NMR (100.6 MHz, $CDCl_3$): δ = 167.5, 152.1, 145.8, 142.9, 133.8, 130.9, 130.4, 127.4, 118.3, 117.1, 111.5, 111.2, 32.0, 29.7. HRMS (ESI-TOF) calcd for $[C_{17}H_{16}N_2O_2+H]^+$ 281.1290, found 281.1291.

Phenyl(1-phenyl-2,3-diazaspiro[5.5]undeca-1,4-dien-3-yl)methanone (**4da**). Following the general procedure, **4da** was prepared from 1-phenyl-2-azaspiro[4.5]dec-1-en-3-ol (**1d**, 115 mg, 0.5 mmol) and benzohydrazide (**2a**, 136 mg, 1.0 mmol); **4da** was isolated as a white powder (83 mg, 50% yield), mp 143-145 °C. ^{1}H NMR (400.1 MHz, $CDCl_3$): δ = 7.80 (d, *J* = 7.6 Hz, 2H, Ph), 7.61 (d, *J* = 7.9 Hz, 1H, CH), 7.40-7.32 (m, 8H, Ph), 5.63 (d, *J* = 7.9 Hz, 1H, CH), 1.74-

1.56 (m, 9H, Cy), 1.17-1.14 (m, 1H, Cy). ^{13}C NMR (100.6 MHz, $CDCl_3$): δ = 168.2, 157.9, 136.5, 133.9, 130.9, 130.3, 128.9, 128.4, 127.9, 127.5, 121.5, 111.6, 38.2, 35.1, 25.5, 20.7. HRMS (ESI-TOF) calcd for $[C_{22}H_{22}N_2O+H]^+$ 331.1810, found 331.1810.

Phenyl(1-(p-tolyl)-2,3-diazaspiro[5.5]undeca-1,4-dien-3-yl)methanone (**4ea**). Following the general procedure, **4ea** was prepared from 1-phenyl-2-azaspiro[4.5]dec-1-en-3-ol (**1e**, 122 mg, 0.5 mmol) and benzohydrazide (**2a**, 136 mg, 1.0 mmol); **4ea** was isolated as a beige powder (65 mg, 38% yield), mp 150-152 °C. ^{1}H NMR (400.1 MHz, $CDCl_3$): δ = 7.79-7.77 (m, 2H, Ph), 7.59 (d, *J* = 7.9 Hz, 1H, CH), 7.41-7.31 (m, 3H, Ph), 7.24 (d, *J* = 7.9 Hz, 2H, Ar), 7.14 (d, *J* = 7.9 Hz, 2H, Ar), 5.61 (d, *J* = 7.9 Hz, 1H, CH), 2.35 (s, 3H, Me), 1.72-1.55 (m, 9H, Cy), 1.21-1.10 (m, 1H, Cy). ^{13}C NMR (100.6 MHz, $CDCl_3$): δ = 168.2, 157.9, 138.3, 134.0, 133.6, 130.9, 130.3, 128.9, 128.6, 127.5, 121.5, 111.6, 38.2, 35.1, 25.5, 21.3, 20.8. HRMS (ESI-TOF) calcd for $[C_{23}H_{24}N_2O+H]^+$ 345.1967, found 345.1968.

3,3-Dimethyl-2-phenyl-4,4a-dihydro-3H,10H-benzo[e]pyridazino[6,1-b][1,3]oxazin-10-one (**5ad**). Following the general procedure, **5ad** was prepared from 3,3-dimethyl-5-hydroxy-2-phenyl-Δ^1-pyrroline (**1a**, 95 mg, 0.5 mmol) and 2-hydroxybenzohydrazide (**2d**, 152 mg, 1.0 mmol); **5ad** was isolated as a beige powder (81 mg, 53% yield), mp 110-112 °C. ^{1}H NMR (400.1 MHz, $CDCl_3$): δ = 8.07 (d, *J* = 7.7 Hz, 1H, Ar), 7.49-7.45 (m, 1H, Ar), 7.42-7.39 (m, 2H, Ph), 7.33-7.31 (m, 3H, Ph), 7.16-7.13 (m, 1H, Ar), 6.99 (d, *J* = 8.2 Hz, 1H, Ar), 5.68 (dd, *J* = 8.2 Hz, *J* = 6.8 Hz, 1H, CH), 2.25-2.23 (m, 2H, CH_2), 1.39 (s, 3H, Me), 1.19 (s, 3H, Me). ^{13}C NMR (100.6 MHz, $CDCl_3$): δ = 160.8, 158.8, 157.0, 136.4, 134.5, 129.3, 128.7, 128.4, 128.0, 123.2, 118.9, 116.3, 82.1, 39.5, 34.2, 27.6, 27.0. HRMS (ESI-TOF) calcd for $[C_{19}H_{18}N_2O_2+H]^+$ 307.1447, found 307.1446.

3,3-Dimethyl-2-phenyl-3,4,4a,5-tetrahydro-10H-pyridazino[6,1-b]quinazolin-10-one (**5ae**). Following the general procedure, **5ae** was prepared from 3,3-dimethyl-5-hydroxy-2-phenyl-Δ^1-pyrroline (**1a**, 95 mg, 0.5 mmol) and 2-aminobenzohydrazide (**2e**, 151 mg, 1.0 mmol); **5ae** was isolated as a beige powder (43 mg, 28% yield), mp 242-244 °C. ^{1}H NMR (400.1 MHz, $CDCl_3$): δ = 8.04 (d, *J* = 7.9 Hz, 1H, Ar), 7.42-7.39 (m, 2H, Ph), 7.33-7.28 (m, 1H, Ar, 3H, Ph), 6.94-6.90 (m, 1H, Ar), 6.72 (d, *J* = 8.1 Hz, 1H, Ar), 5.16 (dd, *J* = 11.0 Hz, *J* = 4.1 Hz, 1H, CH), 4.73 (br s, 1H, NH), 2.13-1.99 (m, 2H, CH_2), 1.38 (s, 3H, Me), 1.12 (s, 3H, Me). ^{13}C NMR (100.6 MHz, $CDCl_3$): δ = 160.5, 159.1, 146.8, 137.1, 133.7, 129.8, 128.7, 128.4, 128.0, 120.2, 117.4, 114.9, 63.6, 40.9, 33.2, 28.2, 28.0. HRMS (ESI-TOF) calcd for $[C_{19}H_{19}N_3O+H]^+$ 306.1606, found 306.1607.

^{1}H and ^{13}C NMR Spectra of 1,4-Dihydropyridazines 4 and Tricycles 5

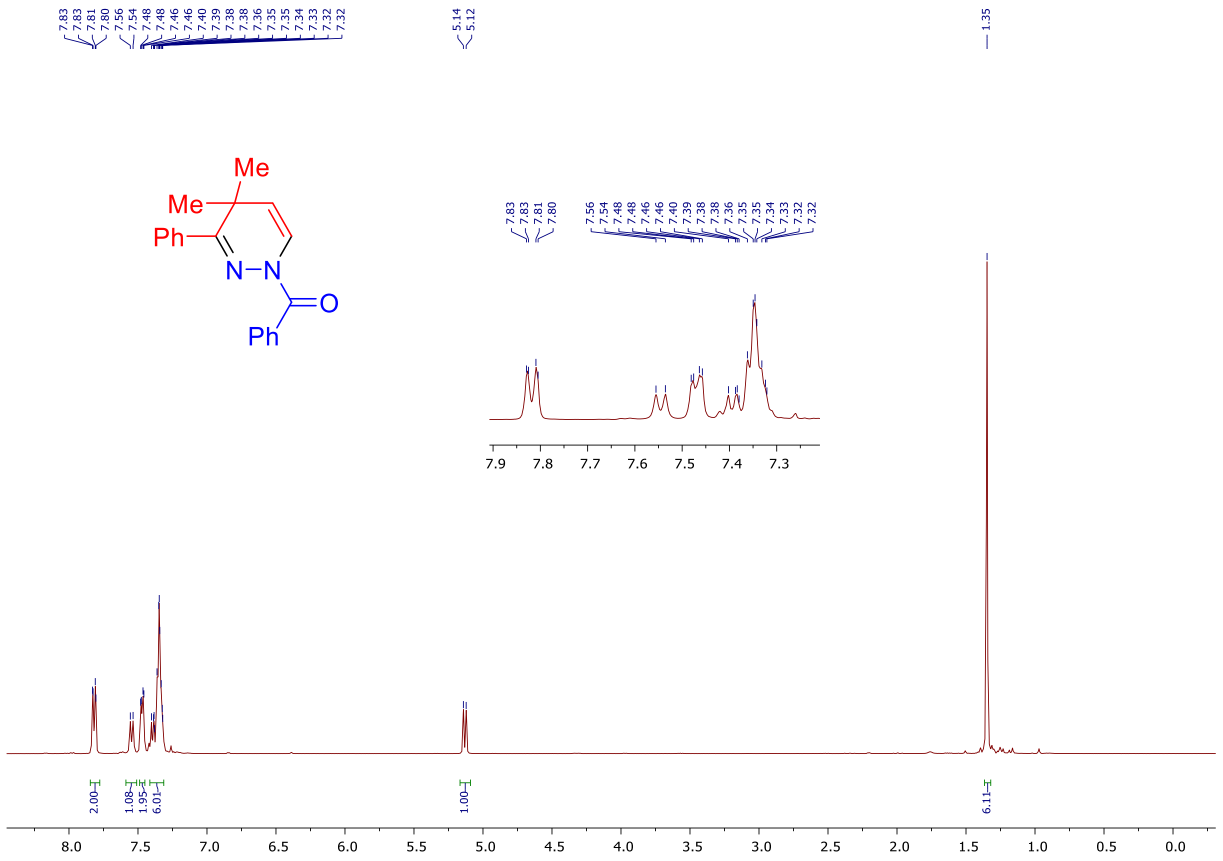

^{1}H NMR spectrum of **4aa** (400.1 MHz, $CDCl_3$)

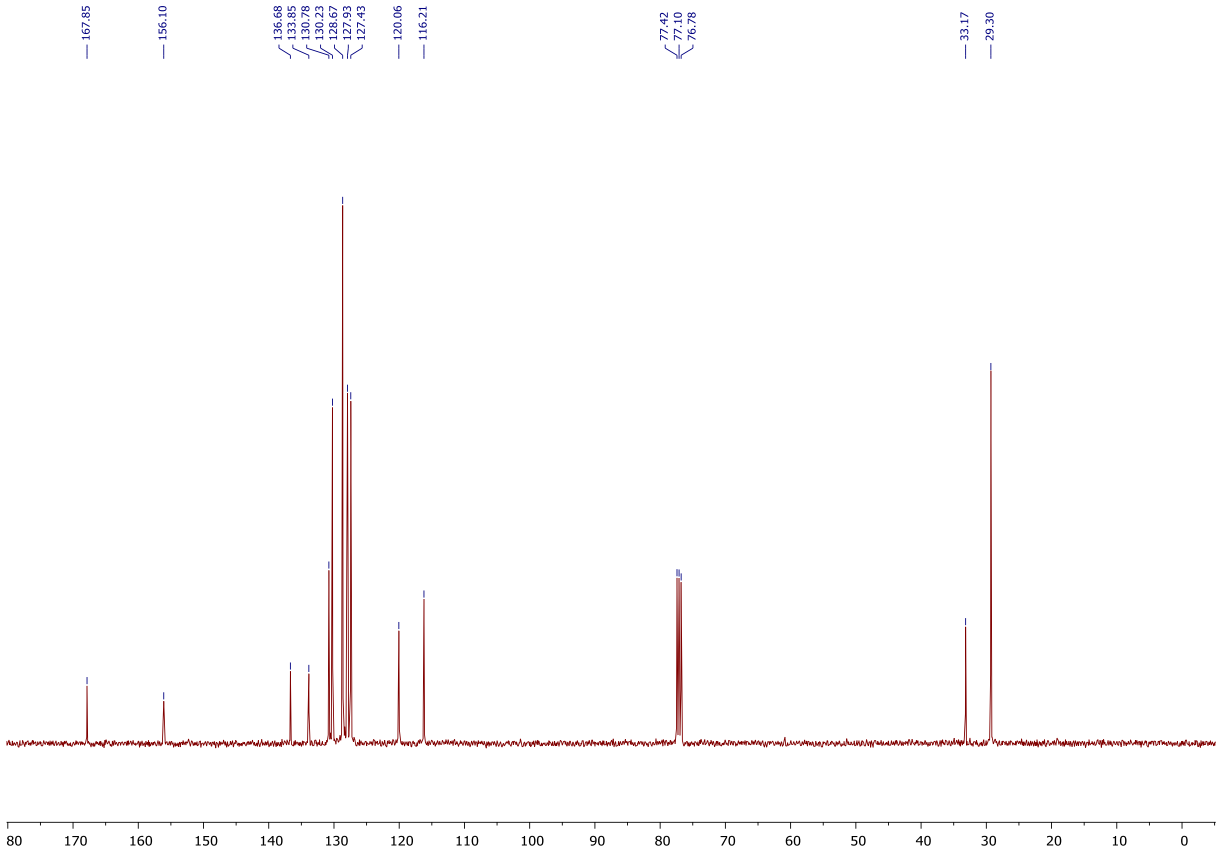

^{13}C NMR Spectrum of **4aa** (100.6 MHz, $CDCl_3$)

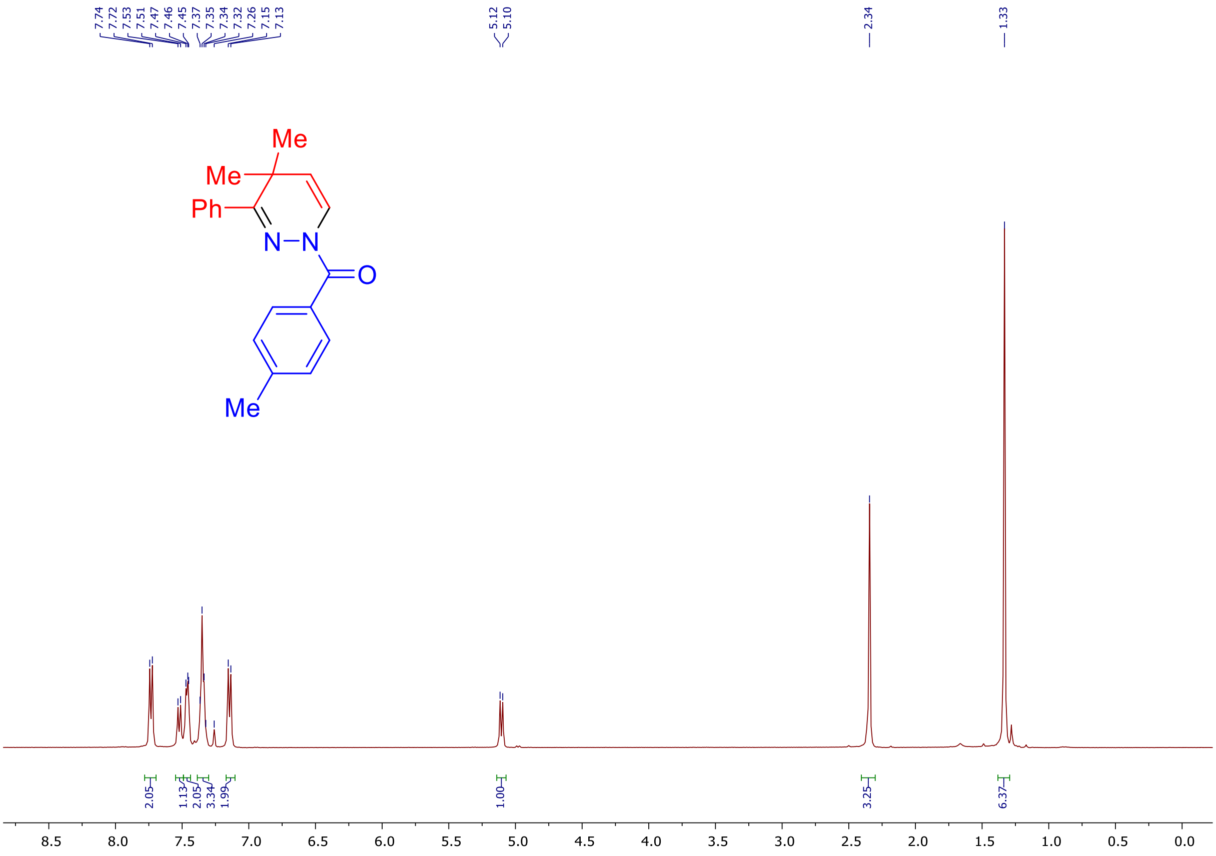

^{1}H NMR spectrum of **4ab** (400.1 MHz, $CDCl_3$)

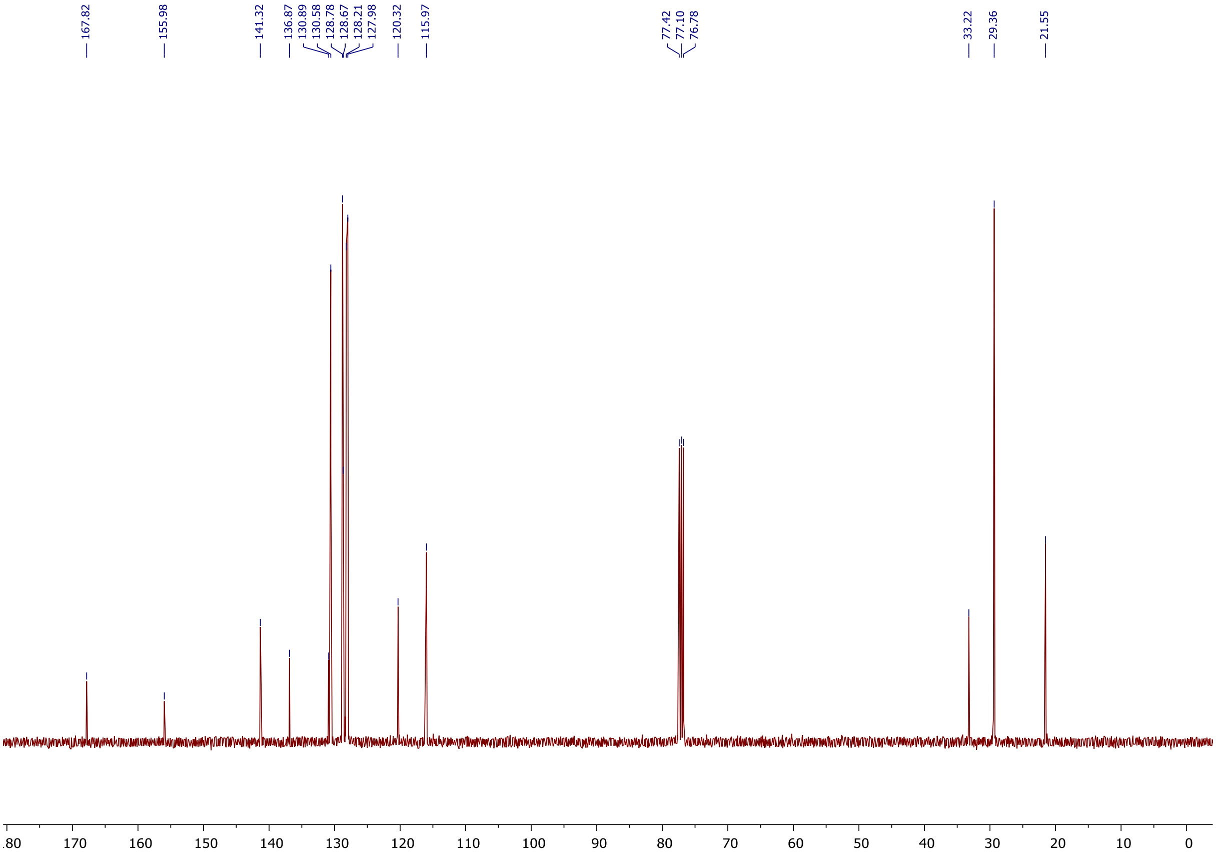

^{13}C NMR spectrum of **4ab** (100.6 MHz, $CDCl_3$)

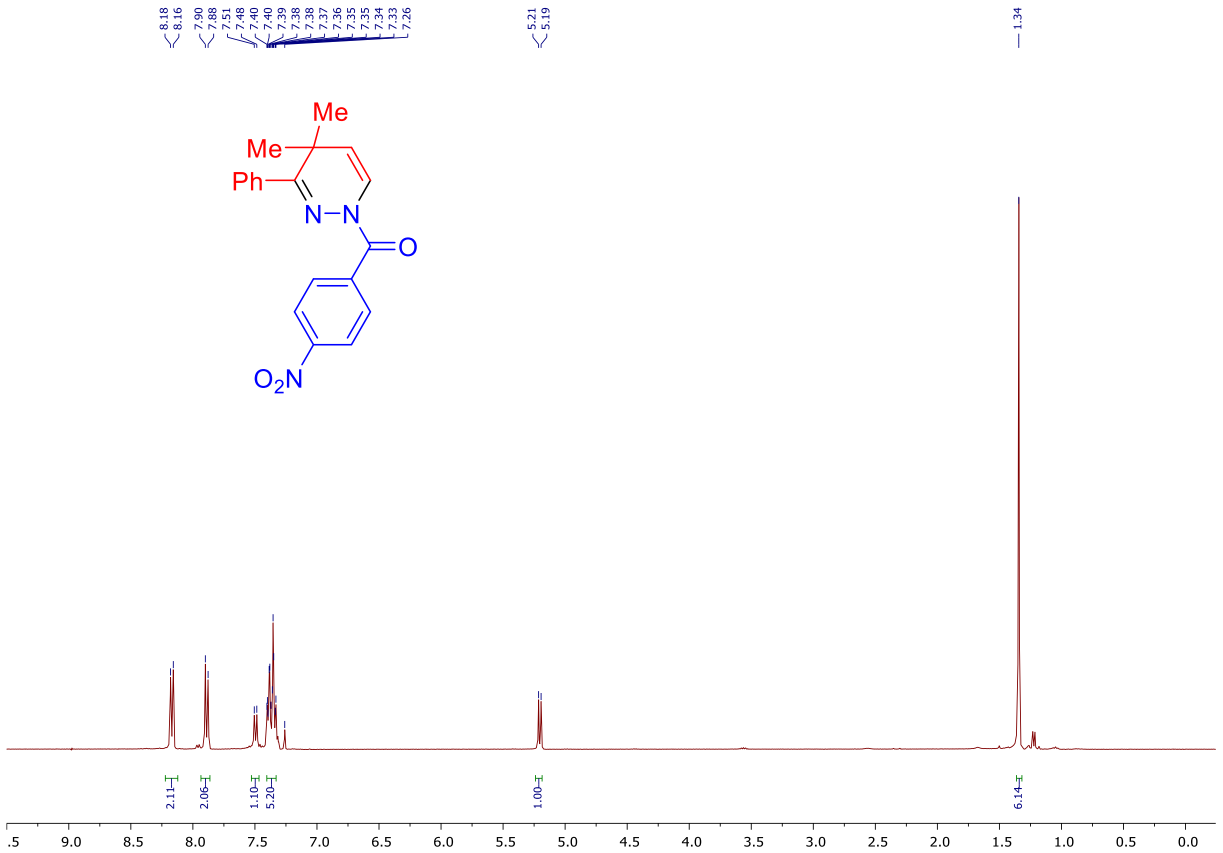

^{1}H NMR spectrum of **4ac** (400.1 MHz, $CDCl_3$)

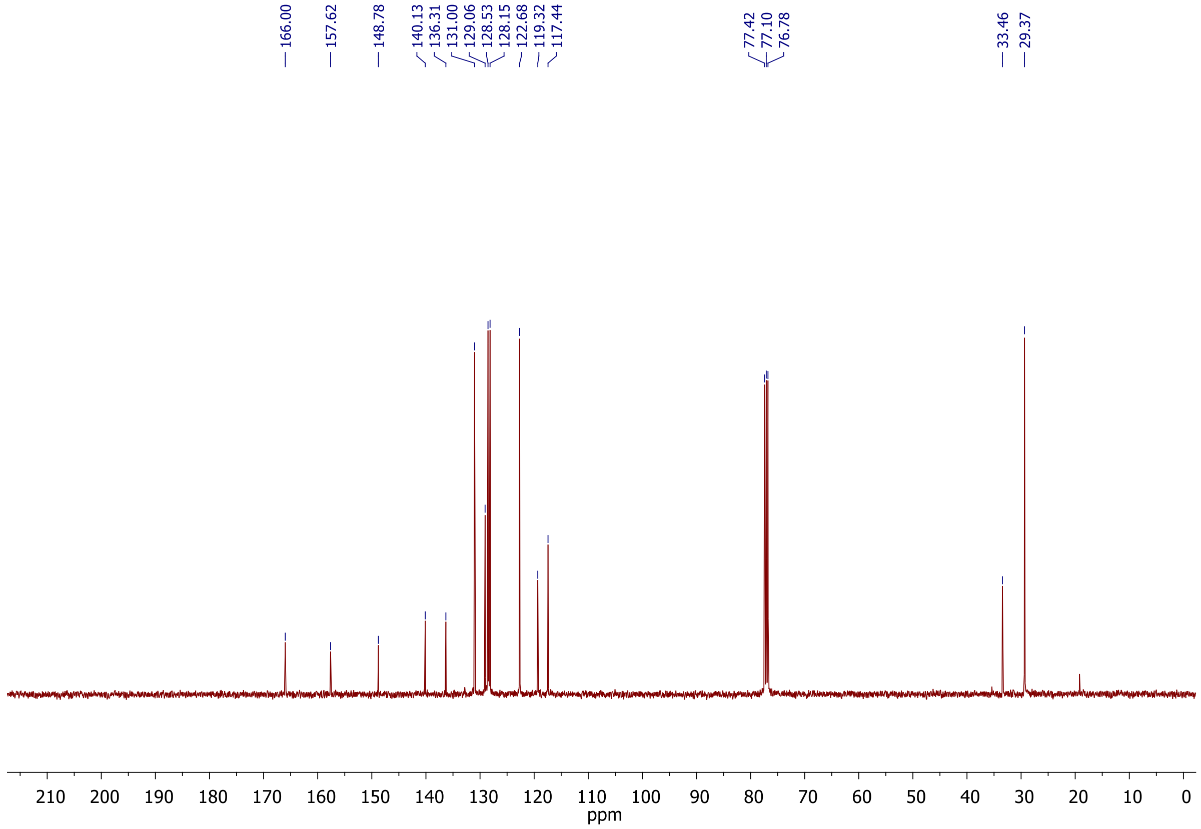

^{13}C NMR Spectrum of **4ac** (100.6 MHz, $CDCl_3$)

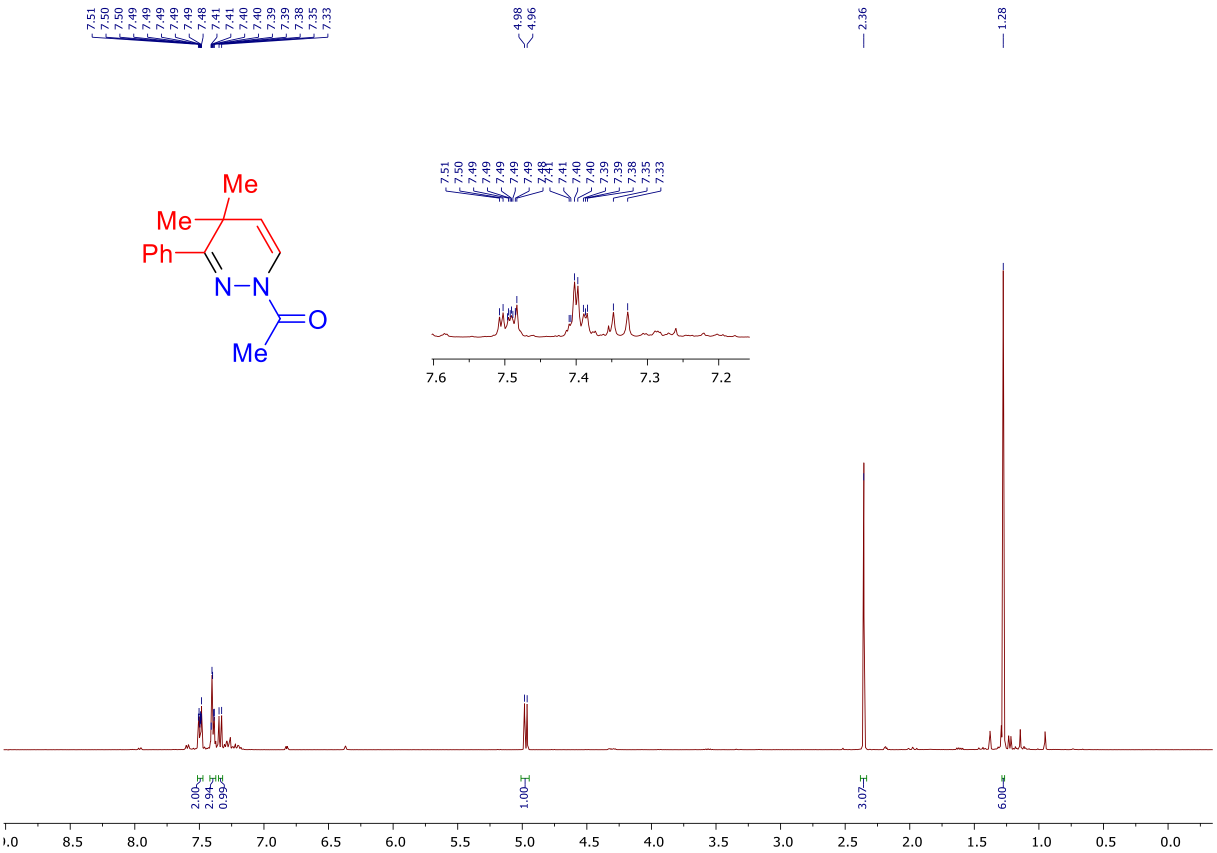

^{1}H NMR spectrum of **4ag** (400.1 MHz, $CDCl_3$)

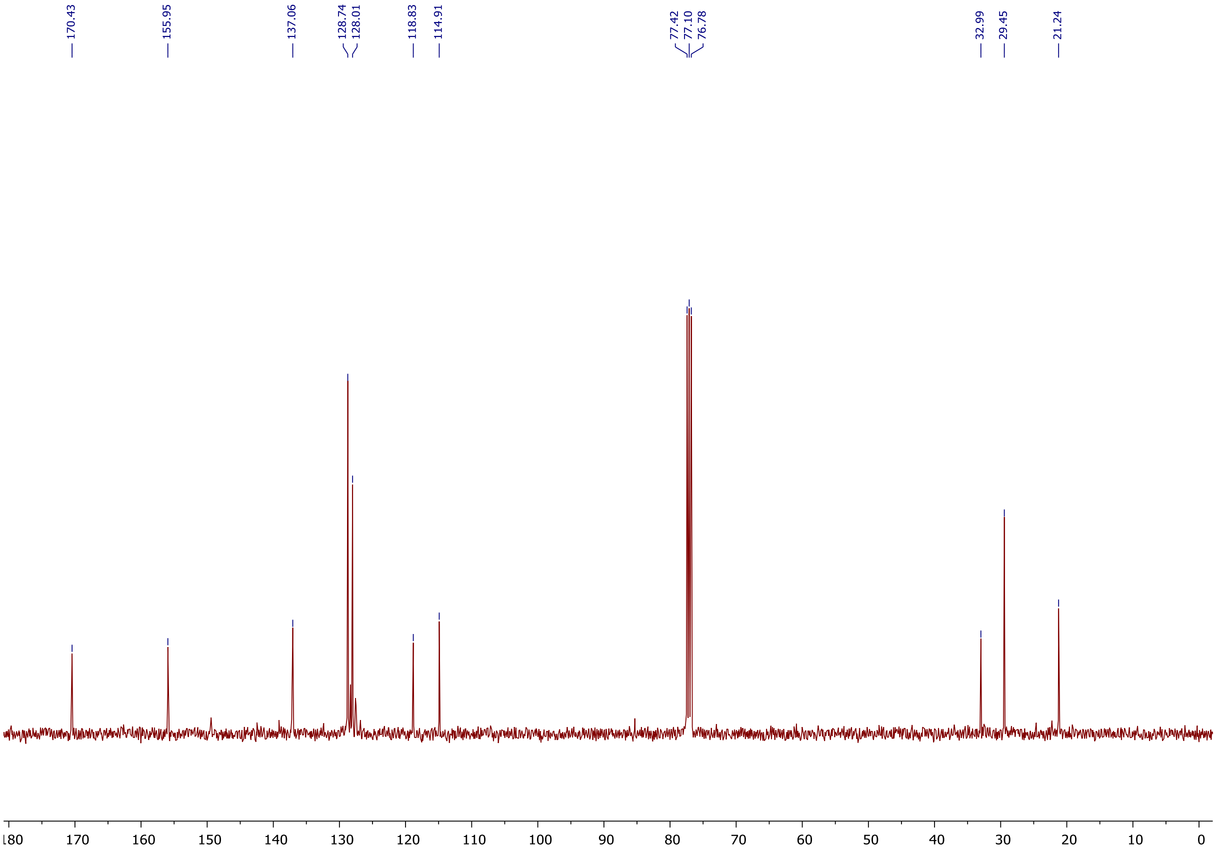

^{13}C NMR spectrum of **4ag** (100.6 MHz, $CDCl_3$)

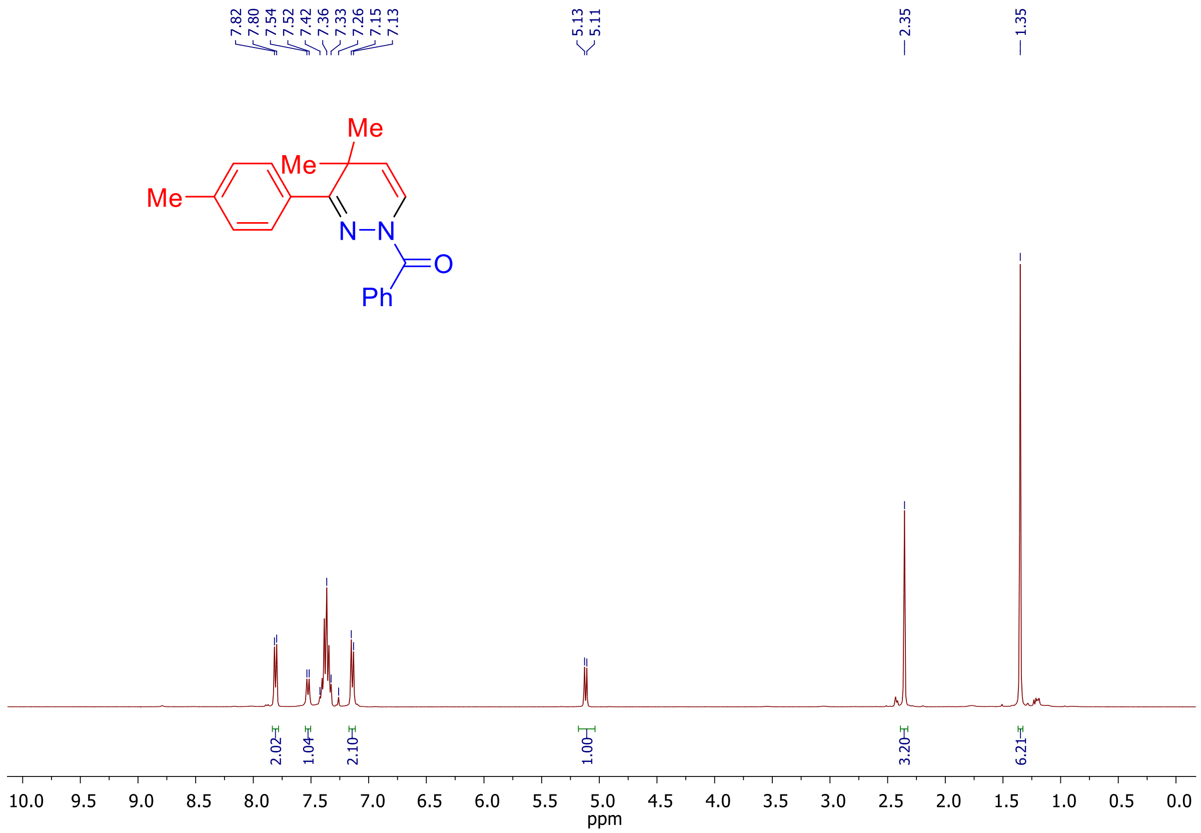

^{1}H NMR spectrum of **4ba** (400.1 MHz, $CDCl_3$)

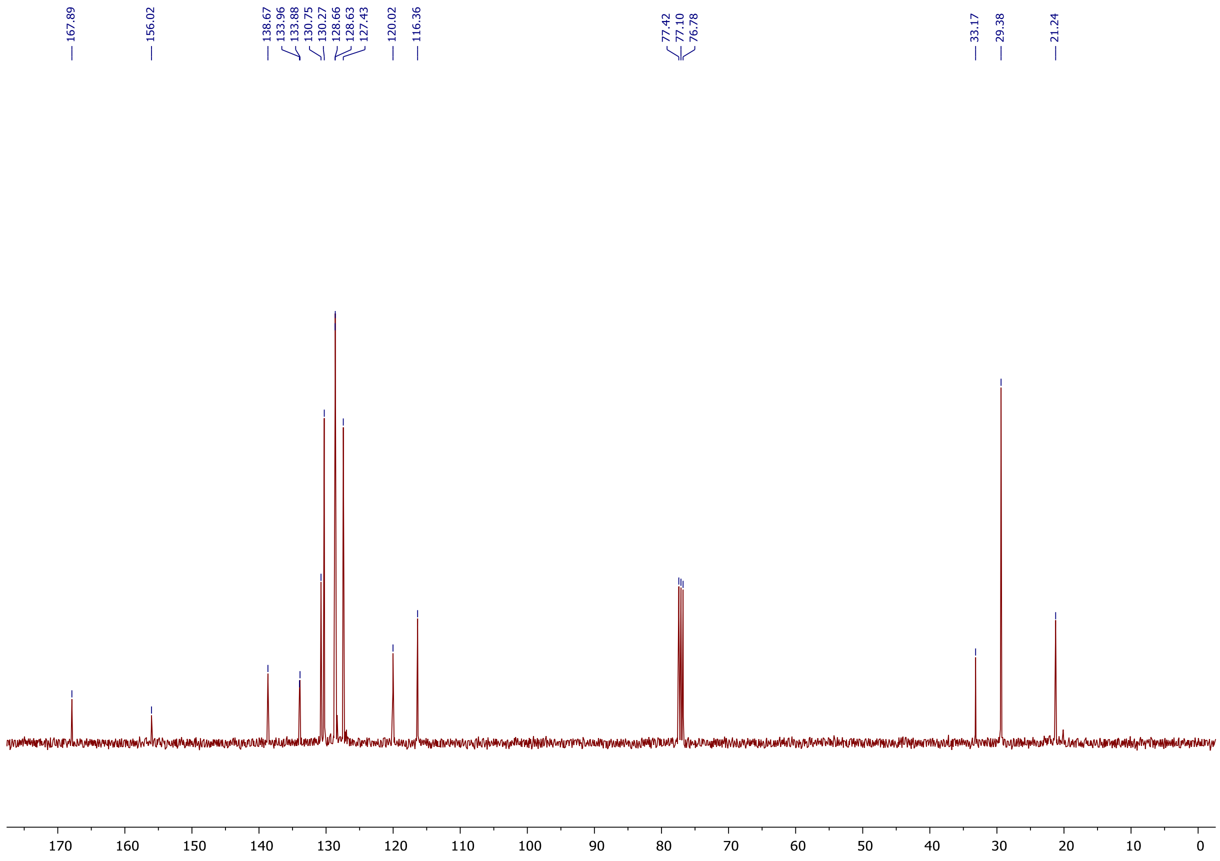

^{13}C NMR spectrum of **4ba** (100.6 MHz, $CDCl_3$)

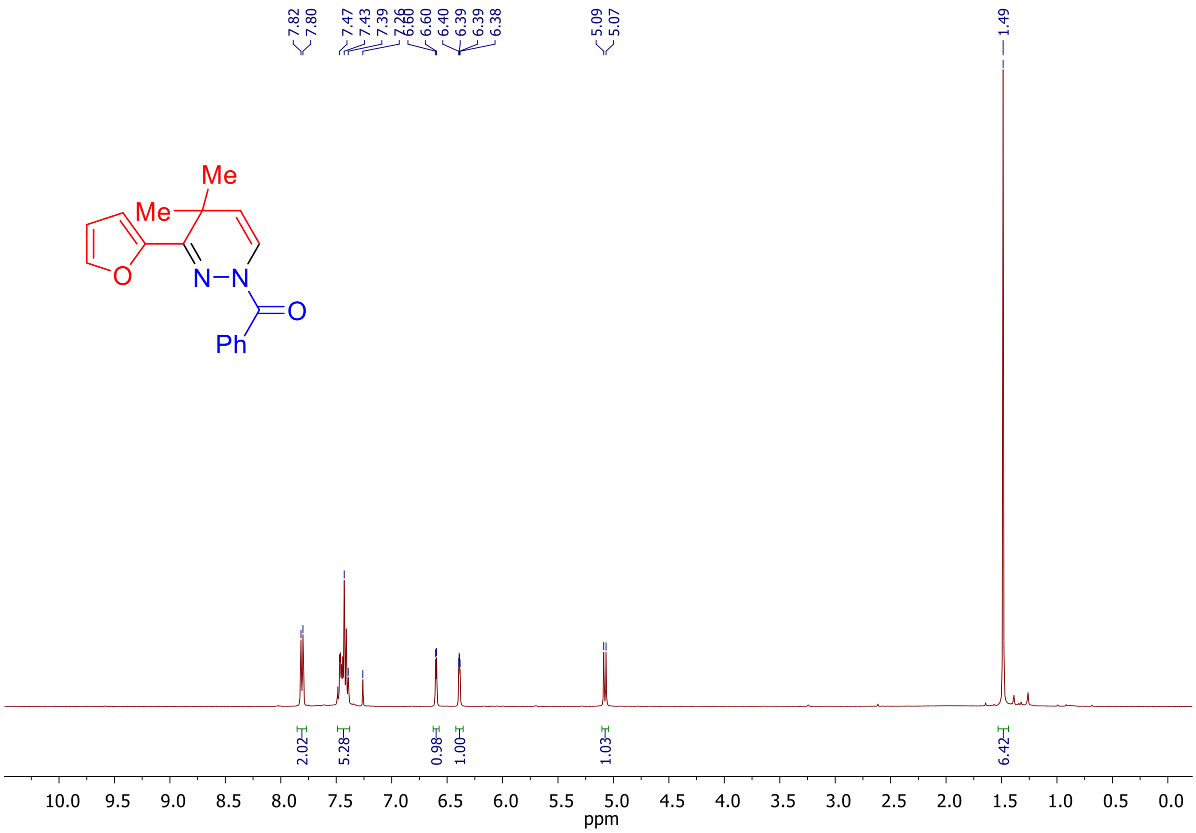

^{1}H NMR spectrum of **4ca** (400.1 MHz, $CDCl_3$)

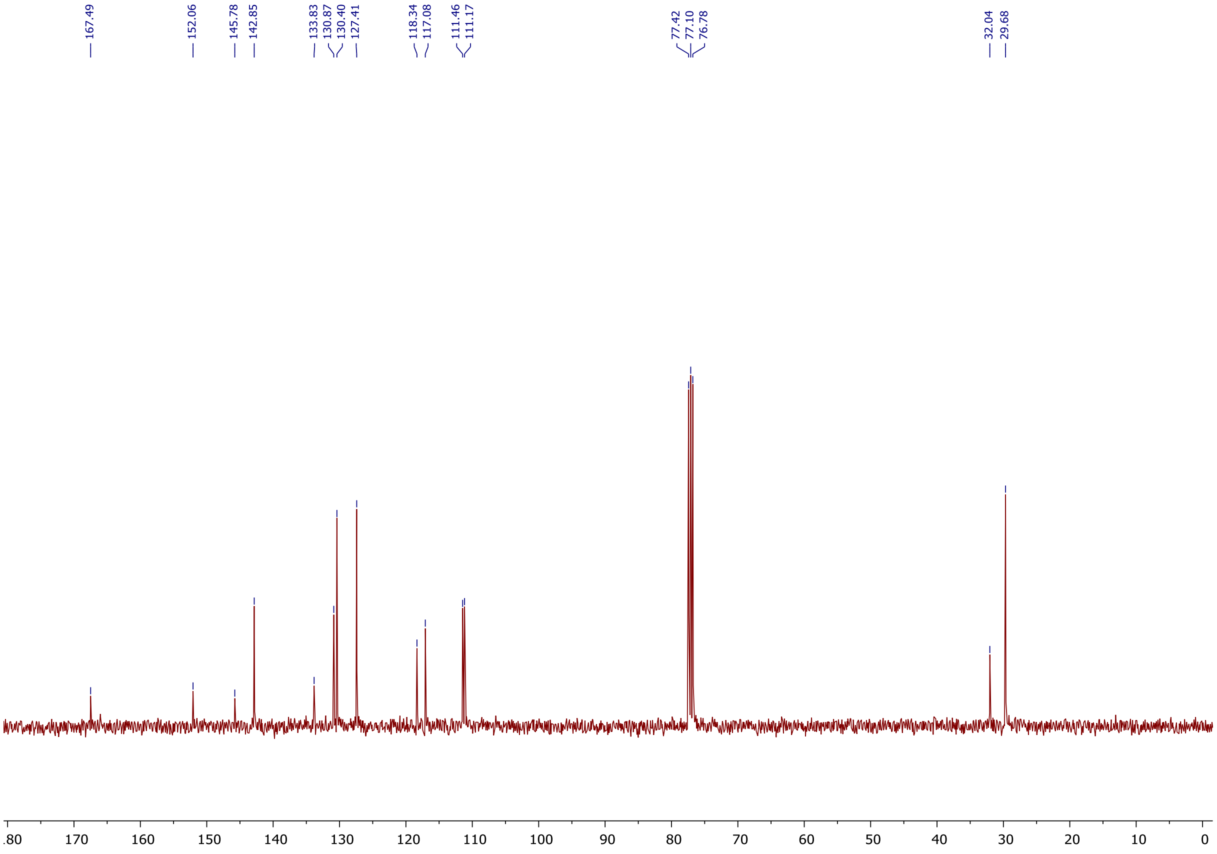

^{13}C NMR spectrum of **4ca** (100.6 MHz, $CDCl_3$)

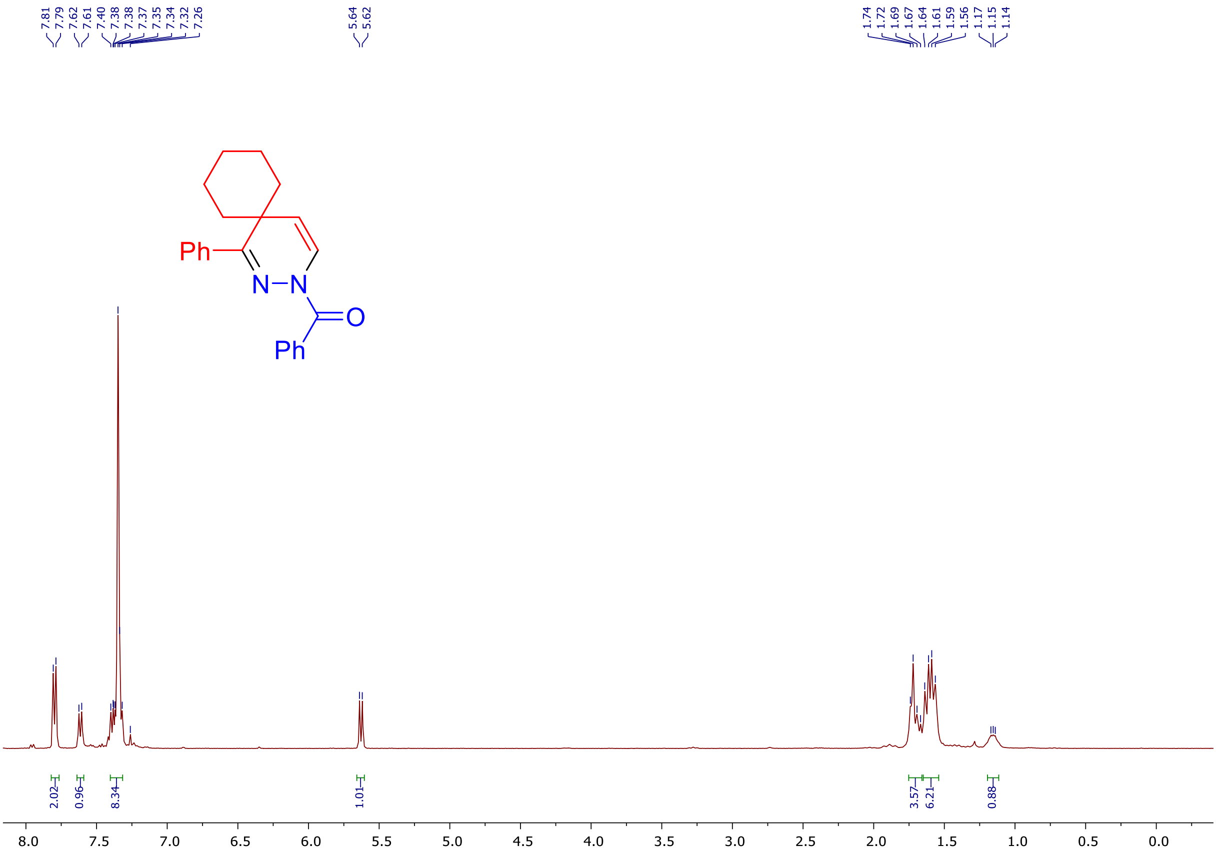

^{1}H NMR spectrum of **4da** (400.1 MHz, $CDCl_3$)

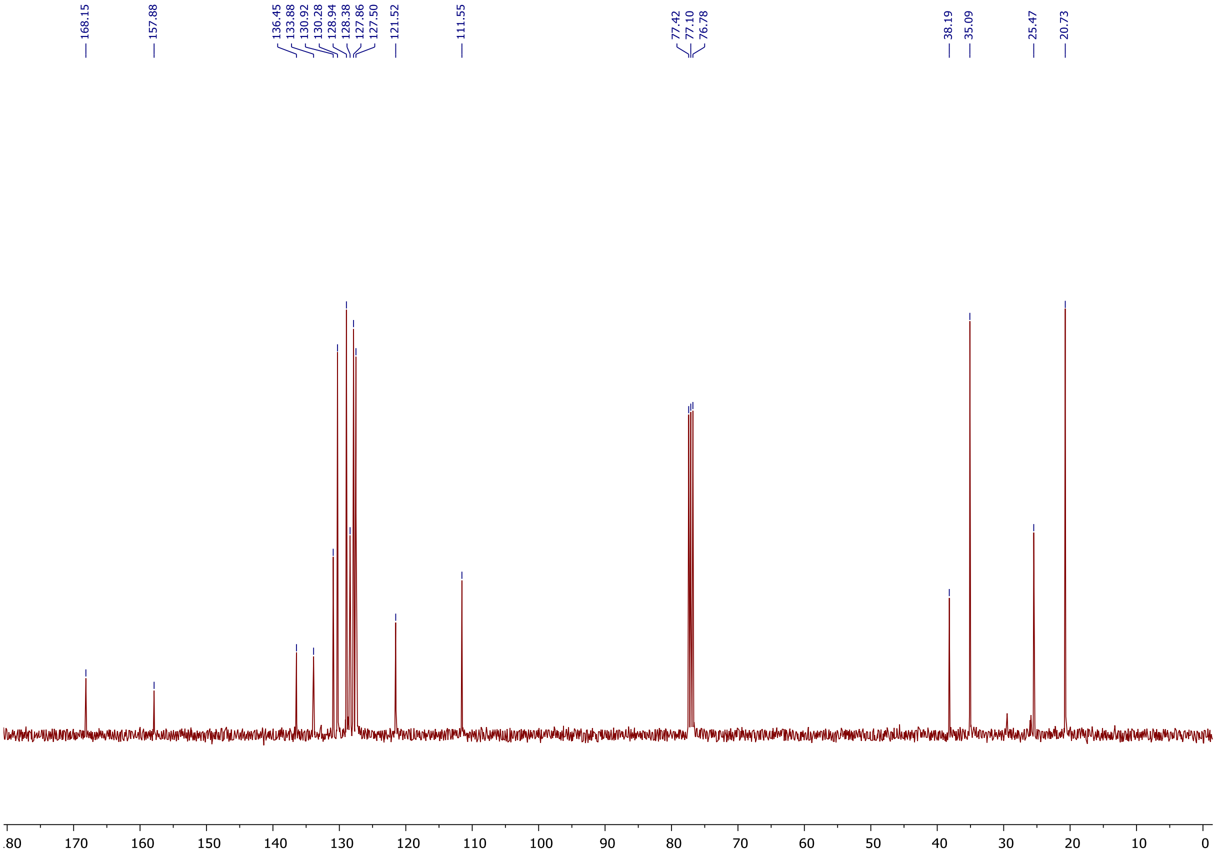

^{13}C NMR spectrum of **4da** (100.6 MHz, $CDCl_3$)

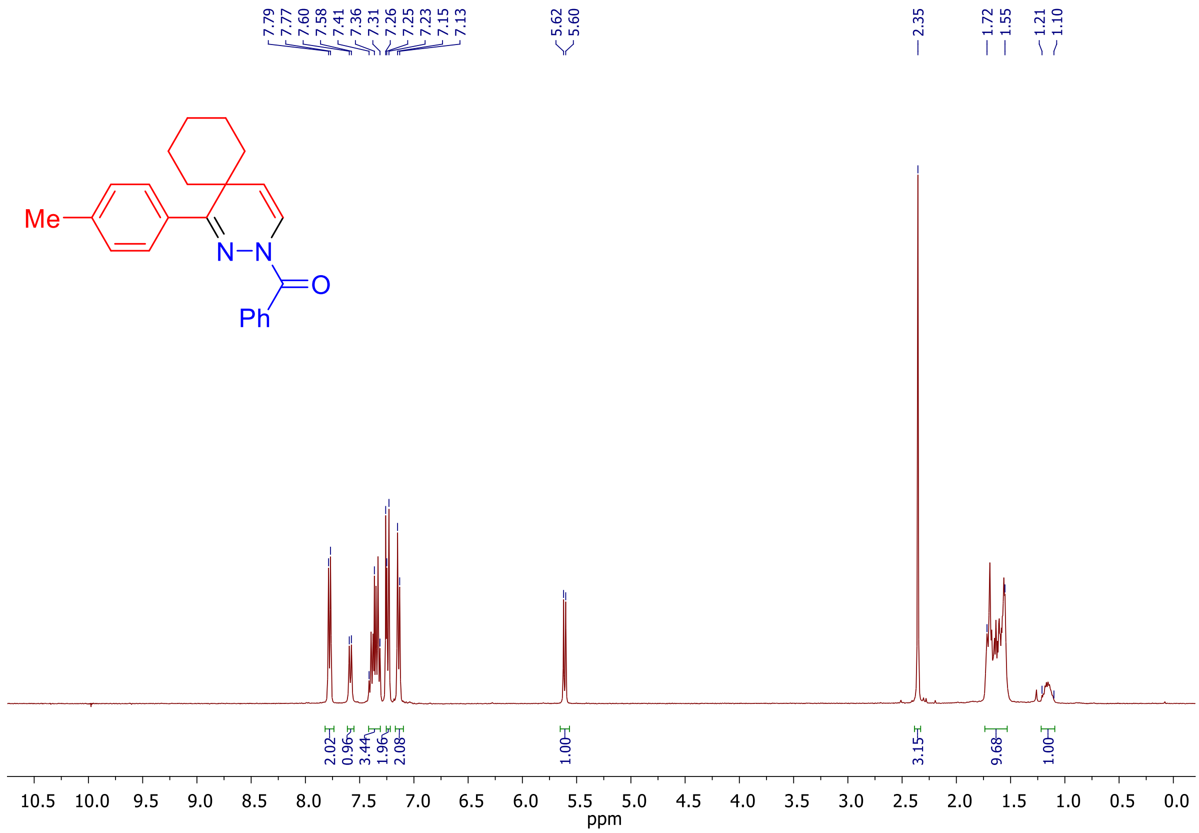

^{1}H NMR spectrum of **4ea** (400.1 MHz, $CDCl_3$)

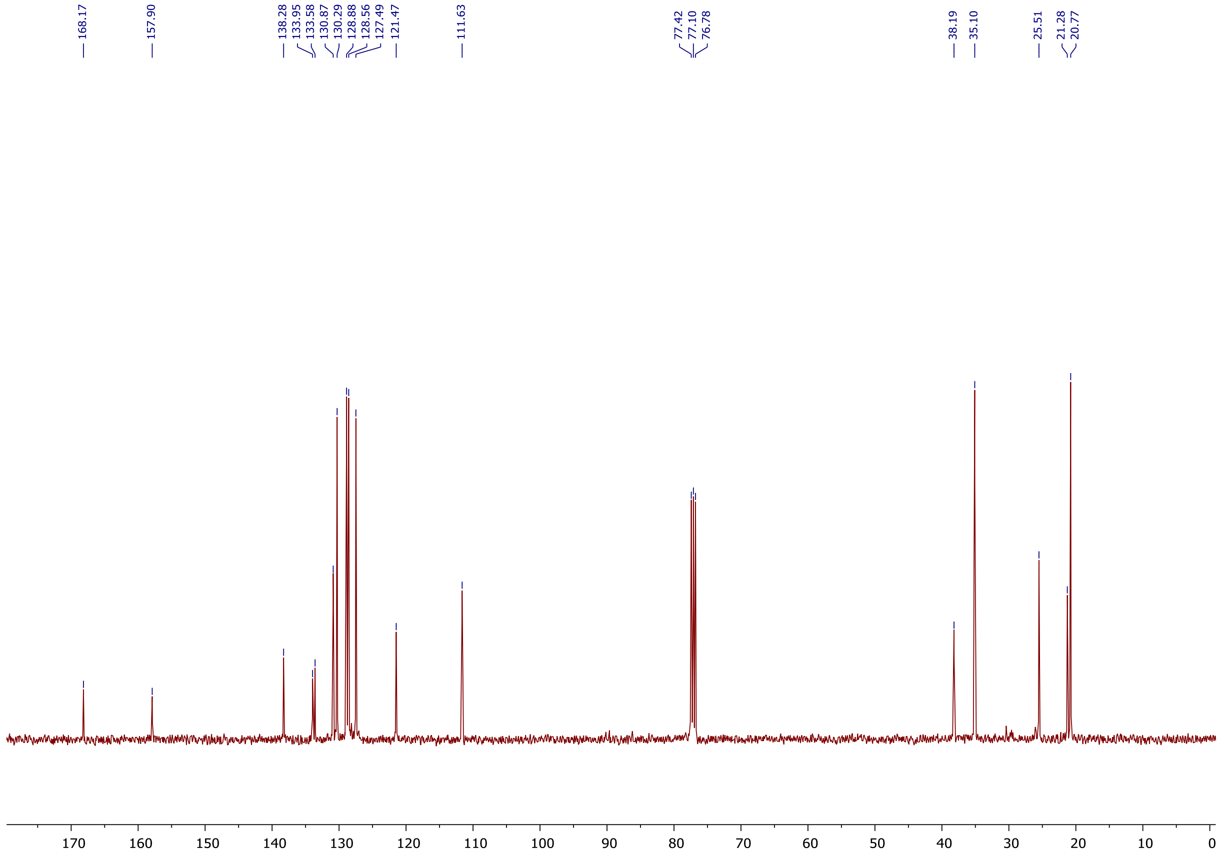

^{13}C NMR spectrum of **4ea** (100.6 MHz, $CDCl_3$)

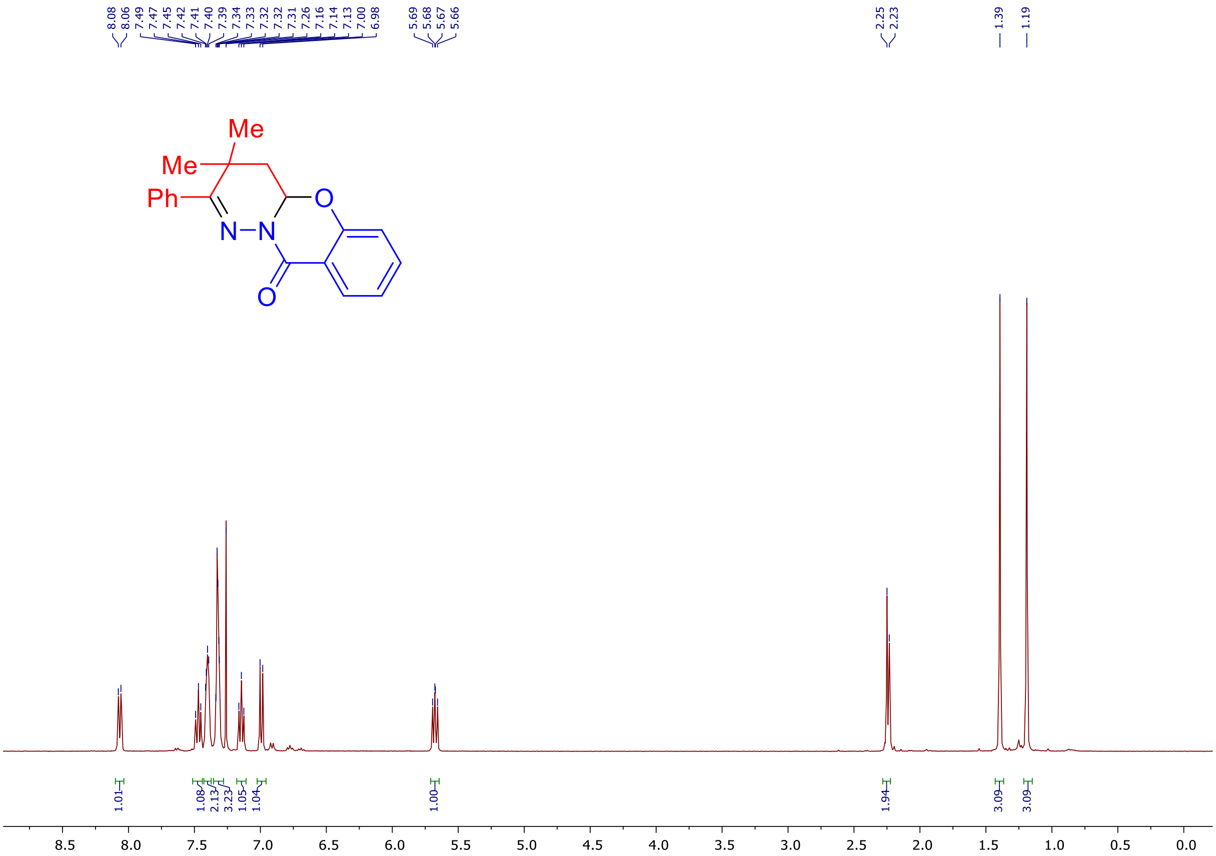

^{1}H NMR spectrum of **5ad** (400.1 MHz, $CDCl_3$)

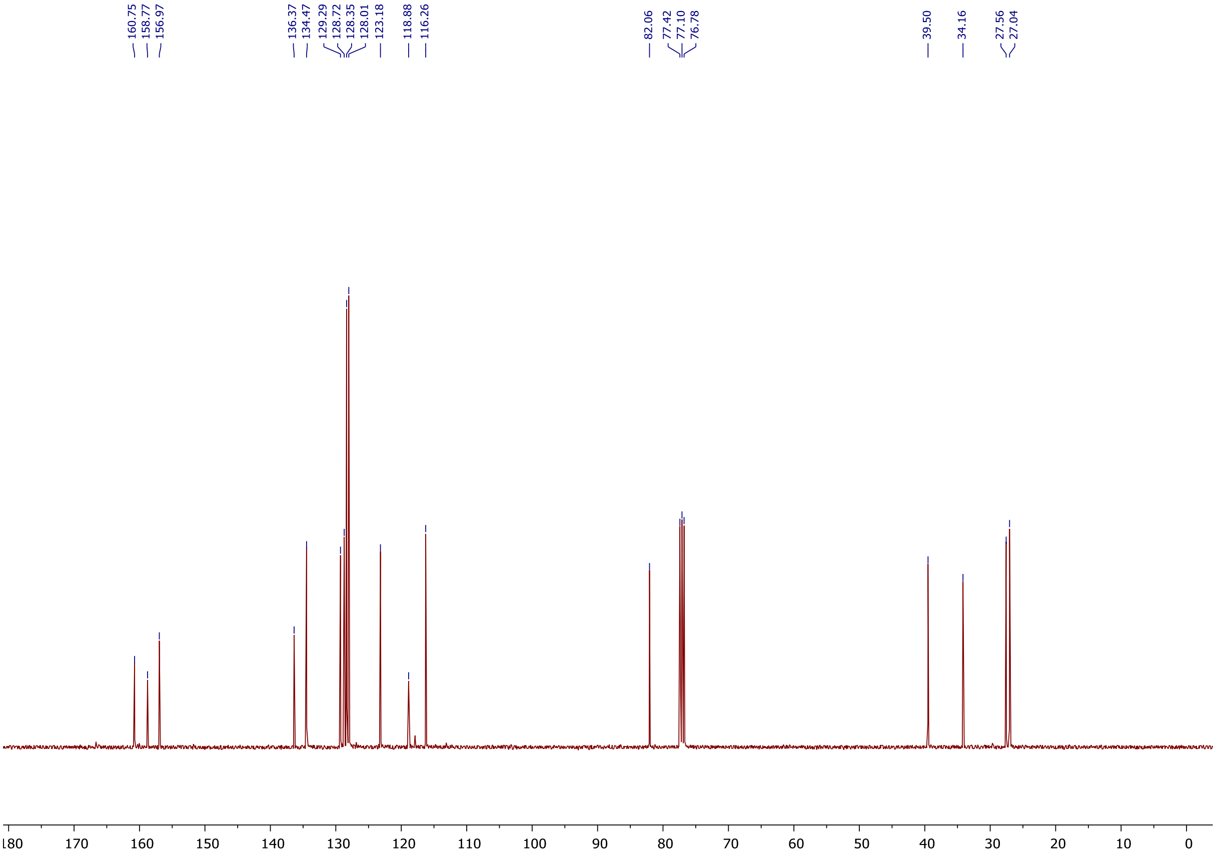

^{13}C NMR spectrum of **5ad** (100.6 MHz, $CDCl_3$)

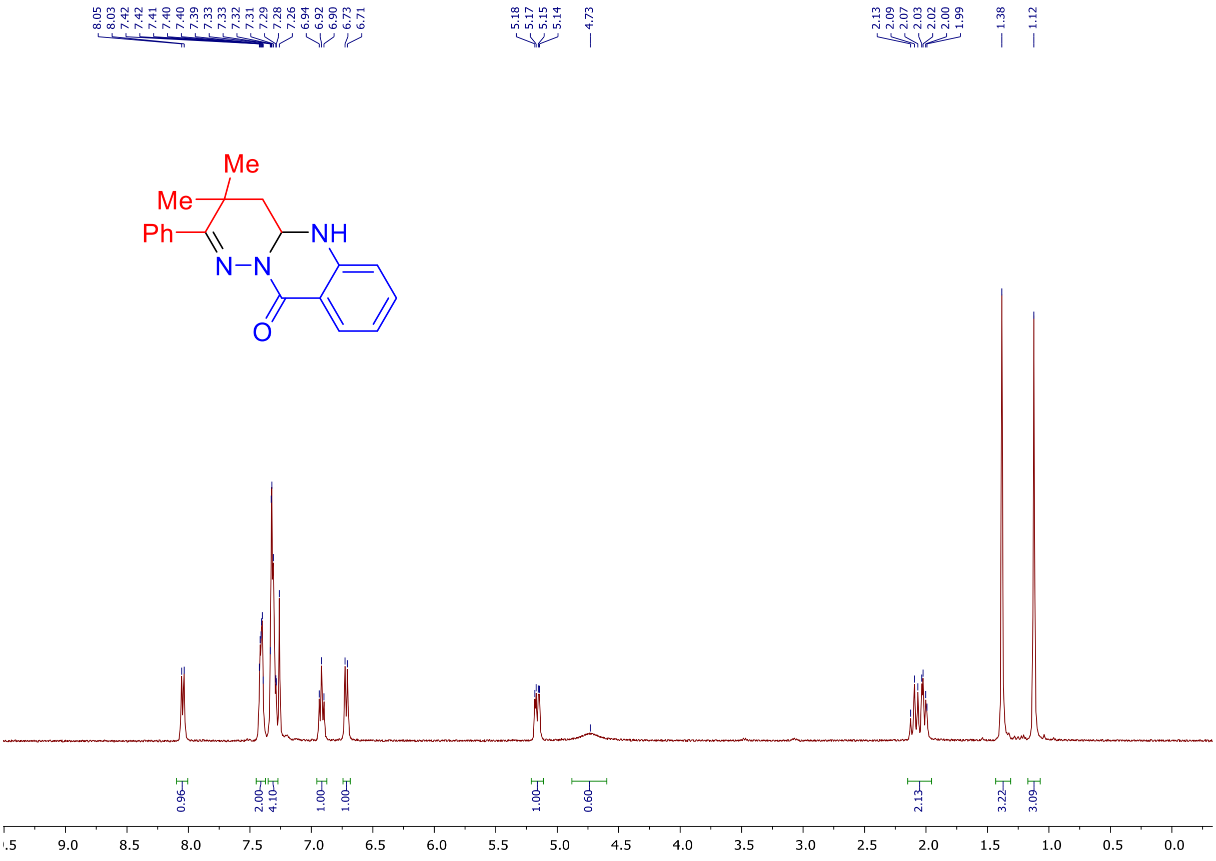

^{1}H NMR spectrum of **5ae** (400.1 MHz, $CDCl_3$)

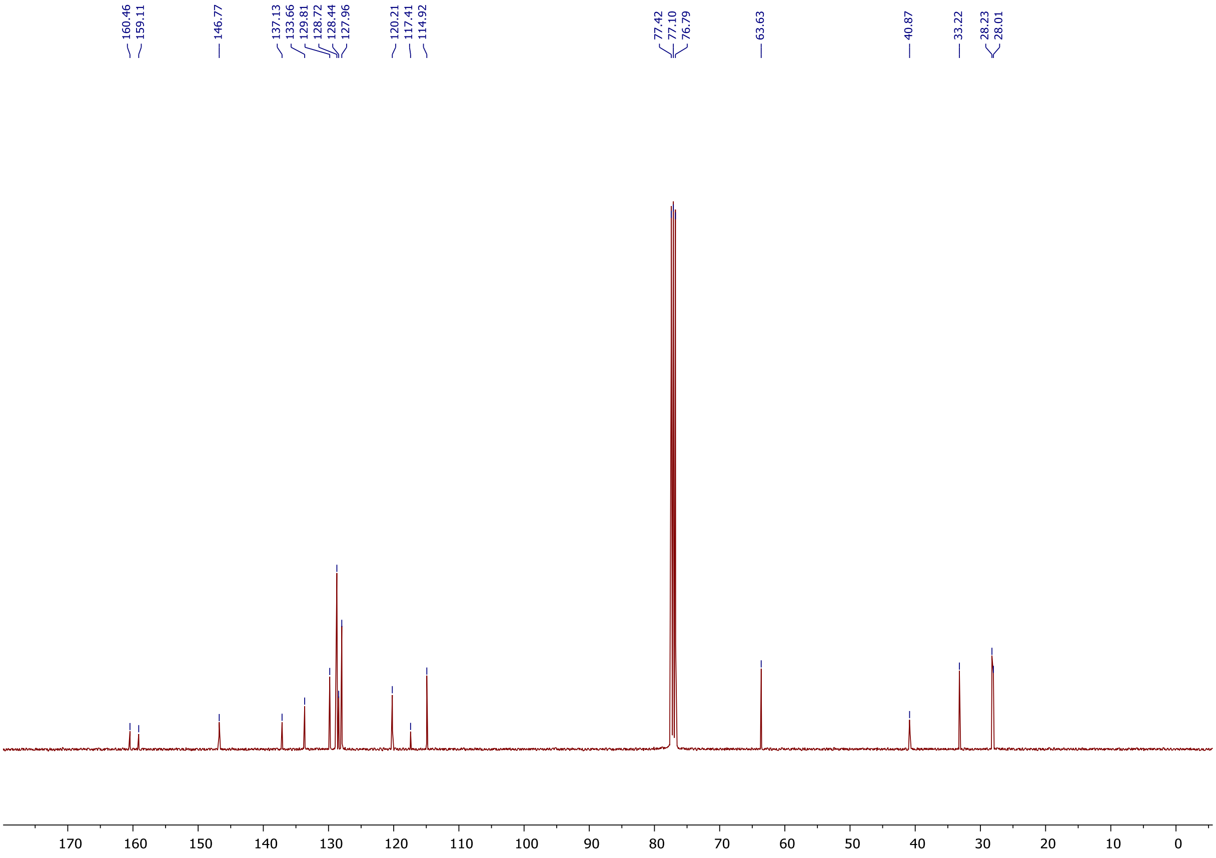

^{13}C NMR spectrum of **5ae** (100.6 MHz, $CDCl_3$)
